# DELLA-NAC Interactions Mediate GA Signaling to Promote Secondary Cell Wall Formation in Cotton Stem

**DOI:** 10.3389/fpls.2021.655127

**Published:** 2021-07-09

**Authors:** Yi Wang, Wanting Yu, Lingfang Ran, Zhong Chen, Chuannan Wang, Yang Dou, Yuanyuan Qin, Qingwei Suo, Yaohua Li, Jianyan Zeng, Aimin Liang, Yonglu Dai, Yiping Wu, Xufen Ouyang, Yuehua Xiao

**Affiliations:** Chongqing Key Laboratory of Application and Safety Control of Genetically Modified Crops, Biotechnology Research Center, Southwest University, Chongqing, China

**Keywords:** gibberellin, DELLA proteins, secondary cell wall formation, NAC proteins, cotton stem

## Abstract

Gibberellins (GAs) promote secondary cell wall (SCW) development in plants, but the underlying molecular mechanism is still to be elucidated. Here, we employed a new system, the first internode of cotton, and the virus-induced gene silencing method to address this problem. We found that knocking down major DELLA genes via VIGS phenocopied GA treatment and significantly enhanced SCW formation in the xylem and phloem of cotton stems. Cotton DELLA proteins were found to interact with a wide range of SCW-related NAC proteins, and virus-induced gene silencing of these NAC genes inhibited SCW development with downregulated biosynthesis and deposition of lignin. The findings indicated a framework for the GA regulation of SCW formation; that is, the interactions between DELLA and NAC proteins mediated GA signaling to regulate SCW formation in cotton stems.

## Introduction

Plant secondary cell walls (SCWs) are particularly important in adopting erect growth and land environment because they provide mechanical support and water transportation tunnels (Kumar et al., 2016). On the other hand, SCWs, comprising most of the plant biomass, are central to generate woods, biofuel, and other industrial uses. In general, SCWs are deposited inside the primary cell wall (PCW) after the cessation of cell expansion. The transition from PCM to SCW deposition encompasses a series of cell and metabolic changes, such as microtubule reorganization, biosynthesis of SCW-specific polysaccharides and lignin, and even activation of SCW-specific cellulose synthase (CESA) genes for cellulose synthesis (Zhong and Ye, 2015; Zhao, 2016; Meents et al., 2018).

In the last decades, a wealth of efforts has been taken to unveil molecular bases that control SCW differentiation and deposition. A conserved pyramid-shaped regulatory hierarchy has been identified to regulate gene expressions required for SCW formation in plants, especially in vascular tissues (Zhong et al., 2008; Nakano et al., 2015; Kumar et al., 2016; Zhao, 2016). This regulatory network contains basically three layers of transcription factors (TFs). In *Arabidopsis*, a group of NAC (NAM, ATAF1/2 and CUC2) family TFs, such as NAC secondary wall thickening (NST) promoting factors 1–3 and vascular-related NAC domains (VNDs) 1–7 (Zhong et al., 2006; Mitsuda et al., 2007; Yamaguchi et al., 2008; Zhou et al., 2014), comprise tier 3 and function as top master switches, which control the transcription of tier 2 TFs, such as MYB proteins (MYBs 46, 55, 61, 83, and 103) and NAC proteins (SND3 and XND1) (Zhong et al., 2010). Tier 2 TFs regulate a number of tier 1 TFs, which directly bind to promoters of structural genes (Hussey et al., 2011; Huang et al., 2015; Kumar et al., 2016). In addition, E2Fc can regulate the transcription of tier 3 TFs and is designated as top-level TF for SCW regulation (Taylor-Teeples et al., 2015). Furthermore, E2FC and tier 3 TFs can also bind to promoters of structural genes and thus promote their expressions as well, and some tier 1 TFs can negatively regulate the expression of tier 3 TFs (Hussey et al., 2011; Huang et al., 2015; Kumar et al., 2016; Zhang et al., 2018).

Gibberellins (GAs) are key plant hormones for many processes of plant development, such as seed germination, stem elongation, leaf expansion, and floral transition (Achard and Genschik, 2009; Daviere and Achard, 2013). DELLA proteins are the major repressor of GA signaling, and GAs generally promote DELLA-repressed downstream activities by mediating their degradation (Daviere and Achard, 2013; Van De Velde et al., 2017). It has been recognized for a long time that GAs promote SCW development, especially the formation of wood (xylem with highly thickened SCW) (Wareing, 1958; Digby and Wareing, 1966; Wang et al., 2013, 2017; Bai et al., 2014; Yuan et al., 2019). The upregulation of GA biosynthesis enzyme genes significantly enhanced SCW (wood) formation in stems of several plants (Israelsson et al., 2003; Biemelt et al., 2004; Park et al., 2015; Jeon et al., 2016). In the process of wood formation, the main roles of GAs are thought to regulate the early stages of wood differentiation, such as cell elongation and expansion (Israelsson et al., 2005; Ye and Zhong, 2015), and xylem differentiation from the cambium (Mauriat and Moritz, 2009). Recently, DELLAs were found to physically interact with a class-I KNOX transcription factor (KNAT1/ BREVIPEDICELLUS) and impair its function in inducing xylary fibers (Felipo-Benavent et al., 2018). In addition, GAs were found to regulate more processes in SCW formation, e.g., cellulose biosynthesis. Huang et al. revealed that a conserved GA-mediated DELLA-NAC signaling cascade regulates SCW synthesis, in which GAs promote cellulose synthesis by releasing SCW master switches NAC29/31 from DELLA and promoting the expression of CESA activator MYB61 in rice (Huang et al., 2015). Another CESA activator, OsMYB103L, could directly interact with DELLA, and GA-mediated DELLA degradation may promote cellulose synthesis and SCW thickening by releasing OsMYB103L (Ye et al., 2015). These results suggested that DELLAs may interact with multiple SCW regulators and form a complex regulatory network for GA signaling in SCW development.

In this study, we employed a new system, the first internode of cotton, to analyze the role of DELLA proteins (GhGAIs) in the GA regulation of SCW development. Yeast two-hybrid (Y2H) and bimolecular fluorescence complementation (BiFC) assays indicated that the cotton DELLA protein interacted with the SCW-related NAC TFs of different tiers, whose knockdown resulted in reduced SCW development in cotton stems. The results suggested that the interactions between DELLA and SCW-related TFs, such as NACs, KNAT1, and MYBs, may form a multiple-knotted regulatory network and mediate GA signaling to promote SCW formation in plant stems.

## Methods and Materials

### Plant Materials and Growth Conditions

Upland cotton (*Gossypium hirsutum* cv Jimian No. 14) plants were used throughout this study and grown in a growth chamber at 25°C under 16 h light (40,000 lux)/8 h dark cycle with 50% humidity conditions.

### Gibberellin and Paclobutrazol Treatment Assays

Gibberellin (GA_4_, Sangon Biotech, Shanghai, China) and paclobutrazol (PAC, Sangon Biotech, Shanghai, China) were dissolved in dimethyl sulfoxide (DMSO) and diluted into water to 100 and 50 μM, respectively. Ten-day-old cotton seedlings were sprayed with GA and/or PAC. The first internode length was measured every 2 days, and the treated plants were collected for phenotype observation and gene expression analyses 8 days later.

### Antibodies and Immunoblotting Assays

To investigate DELLA protein level by immunoblotting, the primary antibody anti-GhGAI1 used in this study was customized at Shanghai Youke Biotechnology Co., Ltd (Shanghai, China). The antibody was generated in rabbits by immunization with 246 amino acids at the N terminal of GhGAI1D. The first internodes from the seedlings were ground in liquid nitrogen and homogenized in three volumes of an extraction buffer (50 mM Tris–HCl, pH 7.5, 150 mM NaCl, 5 mM MgCl_2_, 10% glycerin, 1% Triton X-100, 1 mM DTT, 1× protease inhibitor cocktail). Extracts were centrifuged three times for 10 min at 15,000 × g and 4°C, and the supernatant was quantified by BCA assay. Total soluble proteins were boiled in an SDS sample buffer and further subjected to immunoblotting. The western blots were incubated with a 1:1,000 diluted anti-GhGAI1 antibody.

### Microscopic Observation and Histological Assays

To determine cell length, the epidermis in the middle area of the first internodes were removed with forceps and observed with an optical microscope (Olympus IX81, Olympus, Japan). Cell length was measured in the collected images using ImageJ (http://imagej.net/Fiji). To localize the lignified cell wall, hand-cut transverse sections from the first internodes were incubated in a 1% phloroglucinol solution (in 75% ethanol) for 5 min, treated with concentrated hydrochloric acid for 1 min, and photographed using a stereomicroscope (Zeiss Discovery V20, Carl Zeiss AG, Germany). The autofluorescence of lignin was visualized using an optical microscope (Olympus IX81, Olympus, Japan) under UV conditions.

### Virus-Induced Gene Silencing Assay

To knockdown the expression of *GhGAIs, GhSND2s, GhFSNs*, and *GhVND4s* genes by VIGS, 150–200 bp fragments of *GhGAI1D, GhGAI2D, GhGAI3D, GhGAI4D, GhSND2-1A, GhSND2-2A, GhSND2-3A, GhFSN1A, GhFSN2A, GhVND4-1A, GhVND4-2A, GhVND4-3A, GhVND4-4A*, and *GhVND4-5A* were amplified from the cDNA of developing cotton stems using the VIGS primers (Supplementary Table 1). Asymmetric overlap extension polymerase chain reaction (PCR) methods were employed to fuse related fragments (GhGAI1D-GhGAI2D, GhGAI3D-GhGAI4D, GhSND2-1A-GhSND2-2A-GhSND2-3A, GhFSN1A-GhFSN2A, and GhVND4-1A-GhVND4-2A-GhVND4-3A-GhVND4-4A-GhVND4-5A) (Xiao et al., 2007), which were subsequently inserted into the pTRV2 vector with *Eco*RI and *Kpn*I sites to generate VIGS vectors pTRV2-GhGAI1 and GhGAI2, pTRV2-GhGAI3 and GhGAI4, pTRV2-GhSND2s, pTRV2-GhFSNs, and pTRV2-GhVND4s, respectively. The pTRV1, pTRV2, and derived pTRV2 VIGS vectors were transformed into the *Agrobacterium* strain GV3101 by electroporation (MicroPulser, Bio-Rad, California, United States). The VIGS assays were carried out as described previously (Ma et al., 2016).

### Quantitative Reverse Transcription–Polymerase Chain Reaction Analysis

Total RNAs were extracted from various cotton tissues using a rapid plant RNA extraction kit (Aidlab, Beijing, China). First-strand cDNAs were generated from 1 μg total RNA using a PrimeScript™ RT reagent kit (TaKaRa, Dalian, China) with gDNA eraser to degrade trace genomic DNAs according to the instructions of the manufacturer. Quantitative PCRs were performed with SYBR-Green PCR Mastermix (Vazyme, Nanjing, China). Amplification was monitored on a real-time basis using a CFX96 real-time PCR system (Bio-Rad, California, United States). The thermocycling parameters were as follows: 95°C for 3 min, followed by 40 cycles of 95°C for 5 s, 57°C for 20 s, and a standard melting curve program was added to monitor PCR specificity. GhACT was used as an internal control (Artico et al., 2010). The qRT-PCR results were analyzed using Bio-Rad CFX Manager 2.0 provided by the manufacturer (Bio-Rad, California, United States). The primers are listed in Supplementary Table 1.

### Lignin and Cellulose Contents

The first internodes were dried and crushed into powder and passed through an 80-mesh sieve before component analysis. Lignin content was quantified by a modified protocol (Syros et al., 2004). In brief, a 50-mg sample was weighed, placed in a test tube, and treated with 5 ml 80% (v/v) ethanol in a water bath at 80°C for 2 h. The extracts were centrifuged at 10,000 × g for 10 min, and the supernatants were discarded. Extraction was repeated twice. The pellet was suspended in 4 ml chloroform and incubated for 1 h at 62°C. After centrifuging at 10,000 × g for 10 min, the precipitate was dried for 2 days at 50°C and dissolved in a 2.6 ml acetyl bromide/acetic acid solution (1:3, v/v). After incubation in a 70°C water bath for 1 h, 580 μl of a mixed solution of 17.24% (v/v) 2 N sodium hydroxide and 82.76% (v/v) acetic acid was added, and 100 μl of each sample was transferred to a fresh tube. Finally, 20 μl of 7.5 M hydroxylamine hydrochloride was added to the tube to terminate the reaction, and the volume was fixed to 2 ml with acetic acid. The absorbance at 280 nm was determined using a spectrophotometer (Varioskan LUX multimode microplate reader, Thermo Fisher Scientific, Massachusetts, United States).

Cellulose content was measured using a modified Updegraff method (Updegraff, 1969). Briefly, a 50-mg sample was first treated with 5 ml 80% (v/v) ethanol in an 80°C water bath for 2 h. The mixture was centrifuged at 10,000 × g for 10 min, and the supernatant was discarded. These steps were repeated three times. The precipitate was further digested with a 5 ml acetic/nitric reagent (mixing 150 ml 80% acetic acid and 15 ml concentrated nitric acid) in a boiling water bath for 30 min. After cooling at room temperature and centrifuging at 10,000 × g for 10 min, the supernatant was discarded. The digestion steps were repeated twice until the insoluble pellet became completely white. The pellet was washed twice with 10 ml distilled water and twice with 5 ml acetone, and left to air dry overnight. The dried pellet was re-suspended in 5 ml 72% (v/v) H_2_SO_4_, fully mixed, and let to stand at room temperature for 1 h or more until the pellet was completely hydrolyzed. After centrifuging at 10,000 × g for 10 min, 1 ml supernatant was diluted to 100 ml with distilled water. Then 2 ml dilution were transferred to a fresh test tube, and mixed with 0.5 ml freshly prepared 2% anthrone in ethyl acetate and 5 ml concentrated H_2_SO_4_. After incubation in boiling water for 5 min, the absorbance at 620 nm was measured using a spectrophotometer (Varioskan LUX multimode microplate reader, Thermo Fisher Scientific, Massachusetts, United States). Cellulose concentration was measured against a standard curve.

### RNA Sequencing Analysis

Total RNAs were extracted from the first internode of seedlings using a plant RNA extraction kit (AidLab, Beijing, China). RNA detection, sequencing, and routine data analysis were performed by Shanghai Majorbio Bio-pharm Technology Co., Ltd (www.majorbio.com). Raw data were deposited in GenBank (SRA accession No.PRJNA703439). Paired-end clean reads of 150 nt in size (over 6.29 Gb) were aligned to the revised genome assemble of *G. hirsutum* (https://phytozome.jgi.doe.gov/pz/portal.html#!bulk?org=Org_Ghirsutum_er) using hisat2 v2.1.0. RSEM v1.3.1 was used to count the reads mapped to each gene, and then the number of fragments per kilobase of transcript sequence per million base pairs (FPKM) of each gene was calculated as the normalized expression level (Anders and Huber, 2010). Differential expression analyses were performed using the DEGSeq R package v1.38.0 (http://bioconductor.org/packages/release/bioc/html/DEGseq.html). Genes with more than two fold change and *P* ≤ 0.001 were defined as differentially expressed genes (DEGs). Pathway analysis was mainly based on the Kyoto Encyclopedia of Genes and Genomes (KEGG) database (Kanehisa et al., 2008).

### Yeast Two-Hybrid and Bimolecular Fluorescence Complementation Assays

For the Y2H assay, full length coding sequences of *GhVND1-4D, GhVND4-3A, GhVND4-5D, GhFSN1A, GhFSN2D, GhSND2-1A, GhSND2-1D, GhSND2-2A, GhSND2-2D*, and *GhSND2-3A* were inserted into a pGBKT7 bait vector, and the C-terminal fragments of *GhGAI1A* and *GhGAI1D* (GRAS domain) were cloned into a pGADT7 prey vector. Final bait and prey constructs were co-transformed into yeast strain AH109 according to the manual of the manufacturer (Clontech, Dalian, China). Transformed yeast cells were grown on a SD/-Trp/-Leu medium for selection. Protein–protein interactions were tested on a SD/-Trp/-Leu/-His/-Ade medium.

For the BiFC assay, full-length coding sequences of *GhVND1-4D, GhVND4-3A, GhVND4-5D, GhFSN1A, GhFSN2D, GhSND2-1A, GhSND2-1D, GhSND2-2A, GhSND2-2D, GhSND2-3A*, and *GhGAI1D* were transferred into pEaelyGate201-YN or pEaelyGate202-YC vectors (BioVector, Beijing, China) to generate GhVND1-4D-nYFP, GhVND4-3A-nYFP, GhVND4-5D-nYFP, GhFSN1A-nYFP, GhFSN2D-nYFP, GhSND2-1A-nYFP, GhSND2-1D-nYFP, GhSND2-2A-nYFP, GhSND2-2D-nYFP, GhSND2-3A-nYFP, and GhGAI1D-cYFP. *Agrobacterium* strains (GV3101) containing the above plasmids were co-infiltrated into leaves of *Nicotiana benthamiana*. A yellow fluorescent protein (YFP) fluorescence signal was observed 2 days after infiltration with a confocal microscope (Olympus FV1000, Olympus, Japan). To indicate the nuclei, 4′,6-diamidino-2-phenylindole (DAPI, 1 μg/ml, Sigma-Aldrich, United States) was infiltrated into leaves 30 min before observation.

### Statistical Analysis

All statistical analyses were performed by Student's *t*-test (GraphPad Prism 8.0). The quantitative differences between the two groups of data for comparison with *P* < 0.05 or <0.01) were shown to be statistically significant or extremely significant, respectively.

## Results

### Gibberellin Promotes Stem Elongation and Lignification via DELLA Protein Degradation in Cotton

**Figure 1:**
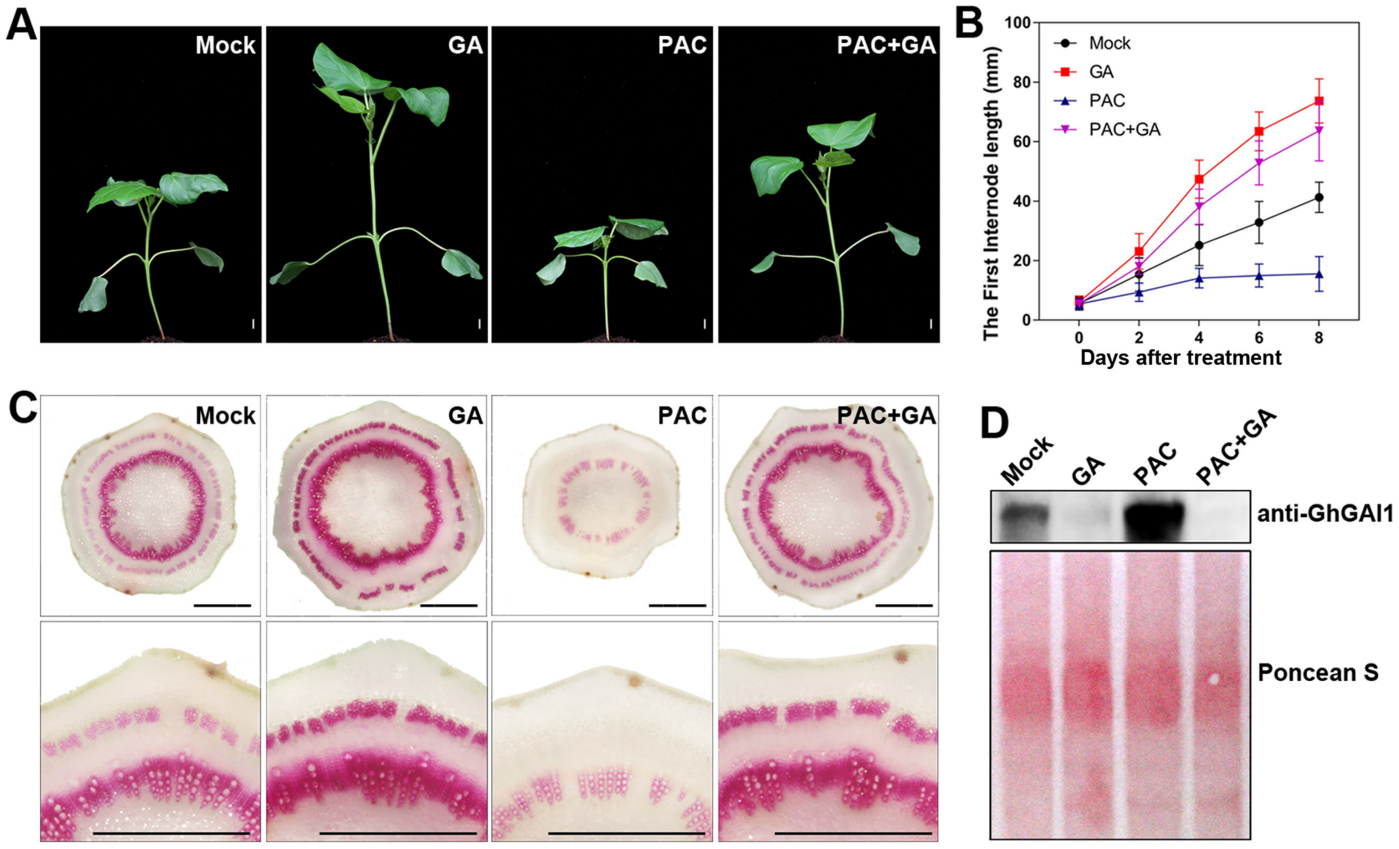
Effects of exogenous GA and PAC on stem elongation and lignification, and DELLA protein level in cotton. Ten-day-old seedlings sprayed with water (Mock), 100 μM GA (GA), 50 μM PAC (PAC), and mixed GA and PAC (GA + PAC) were grown at 25°C under 16 h/8 h (light/dark) conditions for 8 days. **(A)** The phenotype of cotton plants. Bar = 10 mm. **(B)** Time courses of first internode elongation. **(C)** Phloroglucinol staining of cross-sections of the first internode, aligned with the enlarged view at the lower panel. Bar = 1 mm. **(D)** Cotton DELLA protein level detected with anti-GhGAI1 in the first internodes. The amounts of total proteins transferred onto membrane are indicated by Ponceau S staining.

To investigate the roles of the gibberellin signaling pathway during cotton stem development, we treated the cotton seedlings with gibberellin (GA) and/or its biosynthesis inhibitor, PAC. As previously reported (Achard and Genschik, 2009), the GA treatment significantly promoted plant growth and stem elongation, while the PAC treatment showed opposite effects, which can be restored by GA feeding (Figures 1A,B). Next, phloroglucinol staining was employed to detect lignification in the newly developed first internode. It was observed that the GA and PAC treatments significantly increased and decreased the staining area and intensity in xylem and phloem, respectively, compared with the control, indicating that GA enhanced lignification and SCW development in cotton stems (Figure 1C). Furthermore, the immunoblot analysis revealed that the DELLA protein level in the cotton stems was significantly lowered by GA and elevated upon PAC treatment (Figure 1D), indicating that the common GA signaling process, GA-induced DELLA degradation, functioned in the cotton stems. Collectively, these results suggested that GA promoted stem elongation and SCW development via the mediation of the degradation of DELLA proteins in the cotton seedlings.

### Virus-Induced Gene Silencing of GhGAIs Promotes Cotton Growth as Gibberellin Treatment

To genetically analyze the GA function in stem development, we tried to employ the VIGS method to downregulate DELLA genes in the cotton stems. First, we performed a genome-wide analysis of the DELLA gene family in cotton using *Arabidopsis* DELLA protein GAI as a query probe. A total of eight and four DELLA genes were identified in the assembled *G. hirsutum* and extant diploid ancestor species (*G. raimondii* and *G. arboretum*) genomes (Supplementary Table 2), respectively. According to their sequence similarity, cotton DELLA proteins could be divided into four homeologous groups (GoGAI1~4), among which GoGAI1 and GoGAI2 belonged to clade I (DELLA) protein and GoGAI3 and GoGAI 4 belonged to clade II (DGLLA) protein (Supplementary Table 2 and Supplementary Figure 1) (Hu et al., 2011). These genes were named as species abbreviations plus homeologous groups plus subgenome (A or D) in tetraploid (Supplementary Table 2). Based on normalized transcriptomic data in the Cotton FGD collections (https://cottonfgd.org/), we found that clade I DELLA protein genes, especially *GhGAI1A/D*, predominantly expressed in all the tissues investigated, although the expression of clade II DELLA protein genes was also detectable (Supplementary Figure 2).

**Figure 2:**
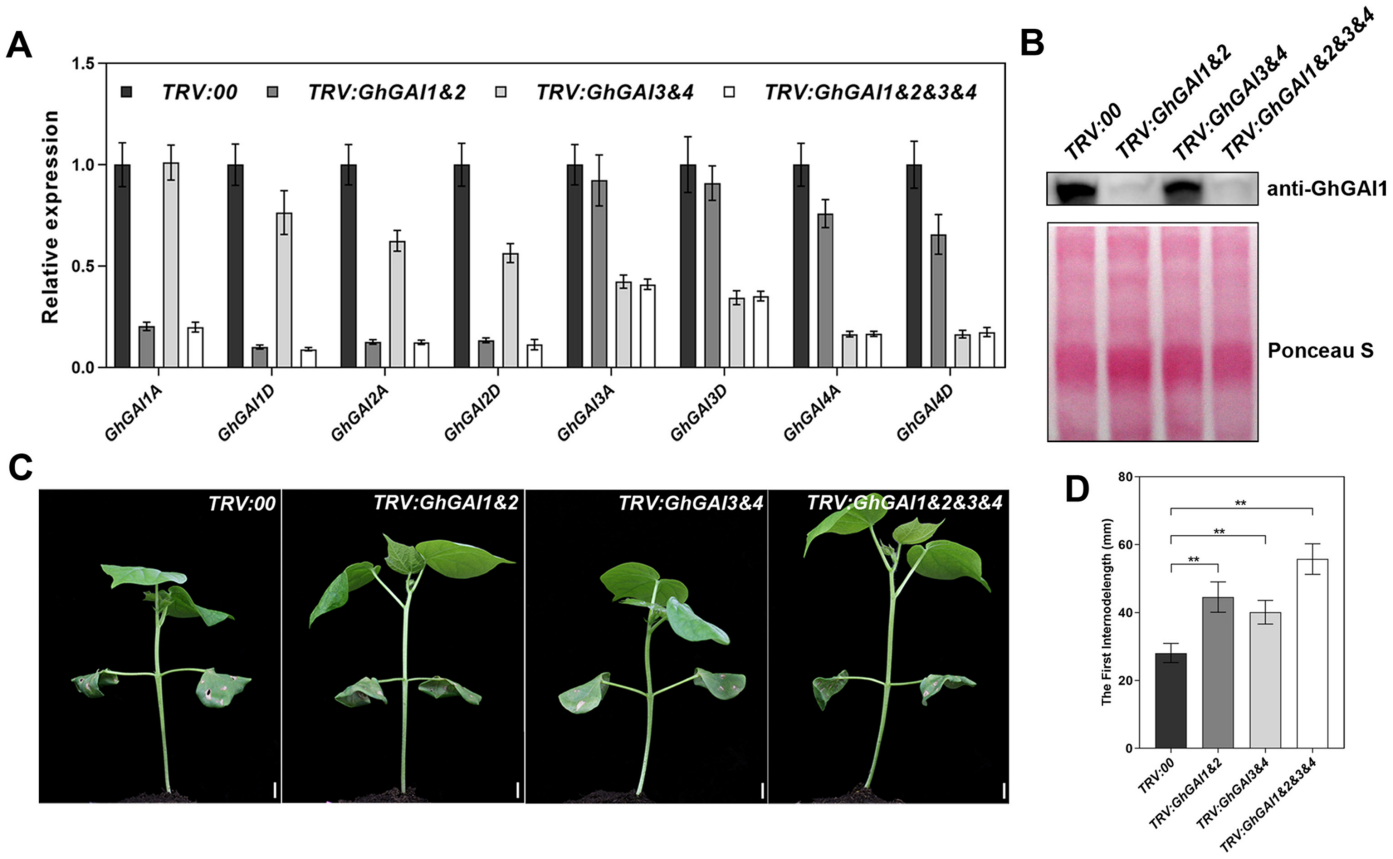
Functional characterization of *GhGAIs* by VIGS analysis. *Agrobacterium* cultures carrying pTRV2-*GhGAI1&2* (*TRV:GhGAI1&2*), pTRV2-*GhGAI3&4* (*TRV:GhGAI3&4*) or both (*TRV:GhGAI1&2&3&4*), mixed with pTRV1 *Agrobacterium*, were infiltrated into two fully expanded cotyledons of 7-day-old cotton seedlings. Empty vector pTRV2-00 (*TRV:00*) was used as control. **(A)** Transcript levels of 8 *GhGAIs* in the first internode were detected 15 days post infiltration (DPI) by qRT-PCR. **(B)** DELLA protein levels detected with anti-GhGAI1 in immunoblot analysis. The amounts of total proteins transferred onto the membrane are indicated by Ponceau S staining. **(C)** Fifteen DPI seedlings. Bar = 10mm. **(D)** The first internode length measured 15 DPI. ** indicates a significant difference compared with control (*t*-test, *P* < 0.01, *n* ≥ 10). Error bars indicate SD.

**Figure 3:**
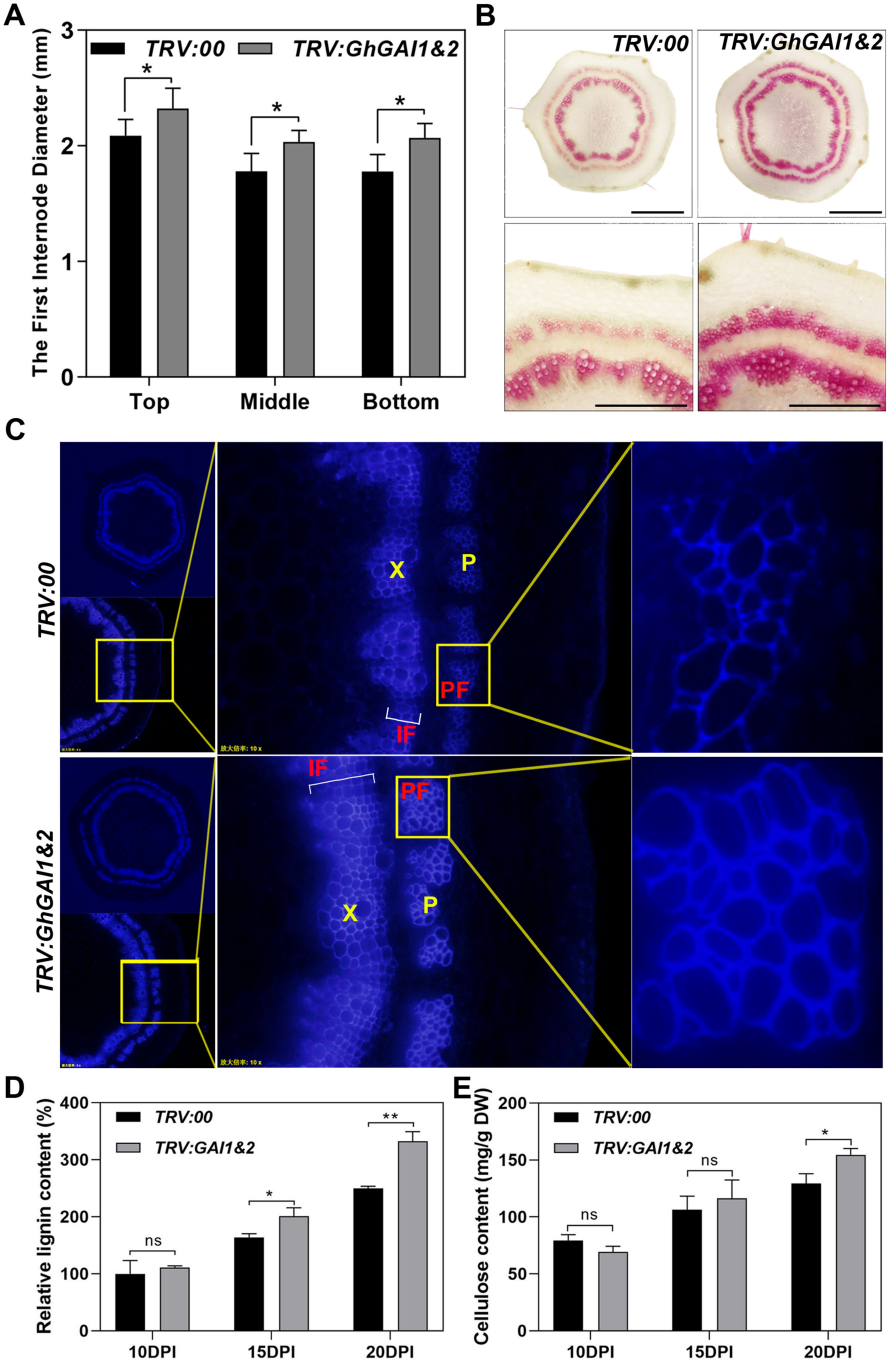
Silencing of GhGAI1 and GhGAI 2 enhances SCW development in the first internodes. *Agrobacterium* cultures carrying empty vector pTRV2-00 (*TRV:00*) or pTRV2-*GhGAI1&2* (*TRV:GAI1&2*), mixed with pTRV1 *Agrobacterium*, were infiltrated into two fully expanded cotyledons of 7-day-old cotton seedlings. **(A)** The first internode diameters at the top, middle and bottom parts measured 20 DPI. * indicates a significant difference compared with control (*t*-test, *P* < 0.05, *n* ≥ 10). Error bars indicate SD. **(B)** Phloroglucinol staining of the cross-sections of 20 DPI first internode in the middle region. Bar = 1 mm or 500 μm (amplification). **(C)** Microscopic observation of cross-sections of 20 DPI first internode in the middle region under UV conditions. **(D)** Lignin and **(E)** cellulose contents in the first internodes, respectively. The internodes were sampled 10, 15, and 20 DPI. Lignin content is shown relative to the control level 10 days post infiltration. * and ** indicate a significant difference compared with the control, with *P*-values of 0.05 and 0.01, respectively (*t*-test, *n* = 3). Error bars indicate SD. P, phloem; PF, phloem fibers; X, xylem; and IF, interfascicular region.

Next, we ask whether the VIGS of GhGAIs may reproduce the effects of the GA treatment on cotton stem development. As shown in Figures 2A,B, TRV:GhGAI1 and GhGAI2 and TRV:GhGAI3 and GhGAI 4, targeting clade I and clade II DELLA genes, respectively, could silence the target genes specifically in the cotton stems. Both GhGAI1 and GhGAI2- and GhGAI3 and GhGAI4-silenced cotton displayed faster plant growth and stem elongation (Figures 2C,D), similar to the effects of the exogenous application of GA. These effects were additive to some extent (Figure 2D), but *GhGAI1&2*-silencing could well-reproduce the effects of the GA treatment, such as lowered DELLA levels, enhanced elongation, and lignification (comparing Figures 2, 3, and Supplementary Figure 3 with Figure 1). Comparing the *GhGAI1&2*-silenced cotton with the control, the diameters of the internodes were significantly increased (Figure 3A) with enhanced SCW development in the xylem and phloem (Supplementary Figure 4). Lignification was significantly enhanced in both vascular bundles and interfascicular regions (Figures 3B,C). It was confirmed that the lignin and cellulose contents in the first internode were significantly elevated in the *GhGAI1&2*-silenced cotton 15 and/or 20 days post infiltration (Figures 3D,E). Collectively, the VIGS of *GhGAIs* (especially *GhGAI1* and *GhGAI2*) could phenocopy the GA treatment to promote stem elongation and SCW formation in the xylem and phloem of the cotton seedlings.

### GhGAI1 and GhGAI 2 Regulate Expression of Secondary Cell Wall-Related Structural Genes in Cotton Stems

To clarify the mechanism of DELLA proteins that regulate secondary cell walls, we carried out RNA-seq analyses using the first internode of a wild type treated with mock, GA, or PAC for 8 days, as well as TRV:00 and *GhGAI1&2-*silenced plants 15 and 20 DPI. A total of 148 differentially expressed genes (DEGs), which responded to GA and PAC in the cotton stems, were identified in *GhGAI1&2*-silenced plants 15 DPI (fold change > 2), including 79 upregulated and 69 downregulated DEGs. Under the same filter conditions, 102 DEGs (47 upregulated and 55 downregulated DEGs) were identified in *GhGAI1&2*-silenced plants 20 DPI (Supplementary Figure 5A). Out of these DEGs, we performed union computation, which resulted in the identification of 97 upregulated and 94 downregulated DEGs as GA signal-responsive genes in the cotton stems, and these DEGs were subjected to subsequent analyses (Supplementary Figure 5B). The KEGG pathway analysis showed that the upregulated genes were mainly involved in phenylpropanoid biosynthesis, starch and sucrose metabolism, galactose metabolism, and zeatin biosynthesis (Supplementary Figure 5C). In contrast, the downregulated genes were enriched in glutathione metabolism and plant hormone signal transduction (Supplementary Figure 5D). Notably, gibberellin 2-oxidase (GA2ox) was upregulated, while gibberellin 3-oxidase (GA3ox) and gibberellin receptor GID1 were downregulated in *GhGAI1&2*-silenced plants, indicating feedback regulation of the gibberellin signal in developing cotton stems.

**Figure 4:**
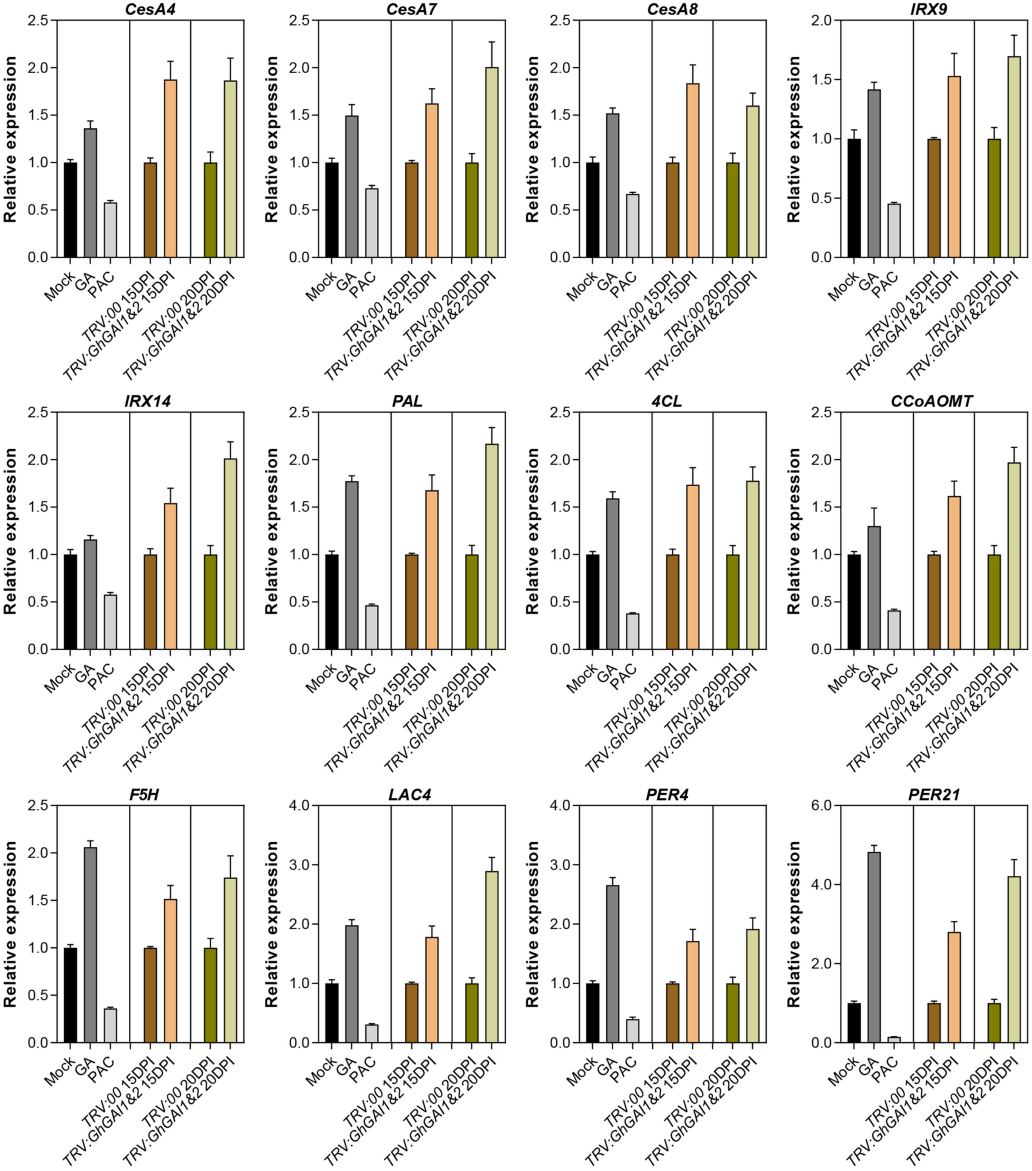
Quantitative RT-PCR analysis of genes related to SCW formation. Total RNAs were isolated from the first internodes. Mock, GA, and PAC represent 10-day-old seedlings treated with water, 100 μM GA, and 50 μM PAC for 8 days. VIGS-treated materials are indicated with pTRV2 vectors (*TRV:00* and *TRV:GhGAI1&2*) plus sampling time (15 or 20 DPI). The relative expression levels in GA/PAC-treated and *GhGAI1&2*-silenced seedlings are then normalized to Mock and *TRV:00*, respectively. Gene accession numbers in cotton FGD: *CesA4* (Gh_D08G0509); *CesA7* (Gh_D05G0079); *CesA8* (Gh_D10G0333); *IRX9* (Gh_A09G1418); *IRX14* (Gh_D11G1966); *PAL* (Gh_D10G2528); *4CL* (Gh_D10G0473); *CCoAOMT* (Gh_A04G1032); *F5H* (Gh_Sca004990G01); *LAC4* (Gh_A11G2936); *PER4* (Gh_A03G0199); and *PER21* (Gh_A09G1415).

We further employed the qRT-PCR method to verify the expression levels of SCW formation-related genes. It was shown that SCW-specific cellulose biosynthesis (*CesA4, CesA7*, and *CesA8*), hemicellulose biosynthesis (*IRX9* and *IRX14*), lignin biosynthesis (*PAL, 4CL, CCoAOMT*, and *F5H*), and polymerization genes (*LAC4, PER4*, and *PER21*) were upregulated in *GhGAI1&2*-silenced plants (Figure 4). The expression levels of these genes were also induced by the GA treatment and reduced by the PAC treatment (Figure 4). These results suggested that DELLA proteins mediated the GA signal to regulate SCW formation by regulating the genes related to cellulose, hemicellulose and lignin biosynthesis, and polymerization.

### DELLA Protein GhGAI1D Interacts With Secondary Cell Wall-Related NAC Proteins

**Figure 5:**
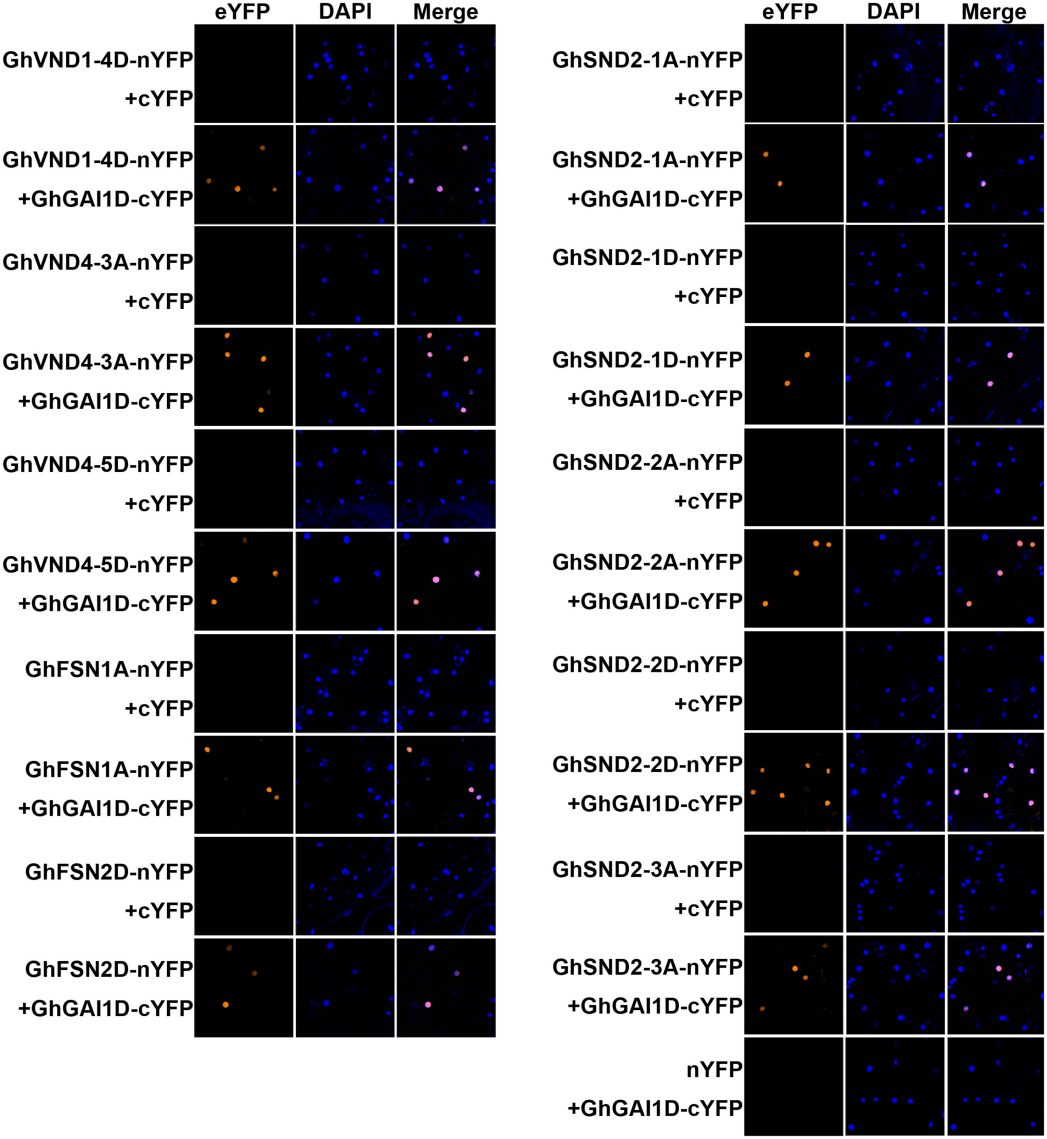
BiFC analyses of interactions between GhGAI1D and SCW-related NACs. NACs fused to N-terminal fragment of YFP (NAC-nYFP) were co-infiltrated with GhGAI1D fused to C-terminal fragment of YFP (GhGAI1D-cYFP) or unfused cYFP into *N. benthamiana* leaves. Unfused cYFP and nYFP were used as negative controls. The nuclei were indicated by DAPI staining.

Next, we attempted to identify any mediator between the GA-DELLA signal and SCW formation in the cotton stems. To this end, we aimed to test the possible interaction between the GhGAI1D and cotton homologs of SCW-related NAC proteins. On the basis of the previous report (Zhang et al., 2018), a total of 62 SCW-related NAC genes were identified in the upland cotton genome (Supplementary Figure 6 and Supplementary Table 3). These genes included 34 homologs of tier 3 NAC TFs (VND1–7, NST1–3) and six SND2/3 homologs, and 18 were homologous to SCW negative regulators (XND1 and VNI2) (Zhao et al., 2007; Yamaguchi et al., 2010). As shown in Table 1 and Figure 5, BiFC analyses showed significant interactions in nuclei between DELLA protein GhGAI1D and all the investigated tier 3 TFs and SND2/3 homologs (each of five NAC proteins), while in the Y2H assay, interactions between GhGAI1D and seven NACs were confirmed, and false-negative results were observed for the rest of the three NACs (Table 1 and Supplementary Figure 7). These results demonstrated that predominant DELLA proteins in the cotton stems (GhGA1D) may have interacted with SCW-related NAC switches of different tiers, implying that these NACs function as mediators of the GA-DELLA signal to regulate SCW formation in cotton stems.

**Table 1:** Interactions between DELLA and NAC proteins.

**Arabidopsis homologs**	**Gene name**	**Gene ID**	Y2H^*^	BIFC^*^
VND1	GhVND1-4D	Gh_D12G0573	Y	Y
VND4	GhVND4-3A	Gh_A12G1990	Y	Y
	GhVND4-5D	Gh_D03G1021	Y	Y
NST1	GhFSN1A	Gh_A12G1049	Y	Y
	GhFSN2D	Gh_D12G2359	N	Y
SND2	GhSND2-1A	Gh_A12G1285	Y	Y
	GhSND2-1D	Gh_D12G1407	Y	Y
	GhSND2-2A	Gh_A05G1339	N	Y
	GhSND2-2D	Gh_D05G1508	N	Y
	GhSDN2-3A	Gh_A06G2028	Y	Y

* *Y and N indicate significant interaction detected and undetected, respectively*.

### Downregulation of DELLA-Interacting NACs Impairs Secondary Cell Wall Formation in Cotton Stem

**Figure 6:**
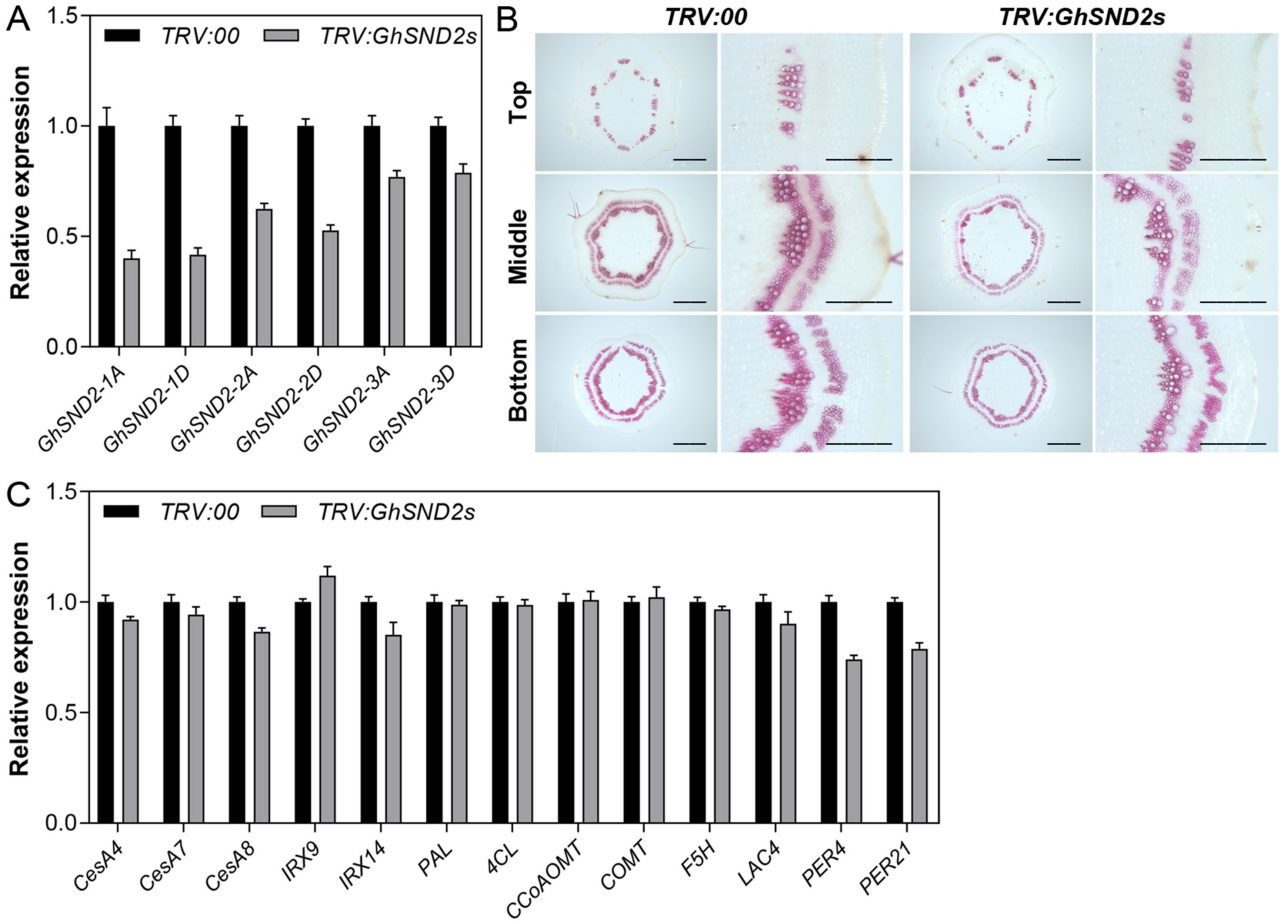
VIGS analysis of GhSND2s. *Agrobacterium* cultures that carry pTRV1 and pTRV2-GhSND2s (TRV:GhSND2s) were infiltrated into two fully expanded cotyledons of 7-day-old cotton seedlings. Empty vector pTRV2-00 (TRV:00) was used as control. **(A)** Transcript levels of six *GhSND2* genes in the first internodes 15 days post infiltration (DPI) by qRT-PCR analysis. **(B)** Phloroglucinol staining of the cross-sections of 15 DPI first internodes. Bar = 1 mm or 500 μm (amplification). **(C)** Quantitative RT-PCR analysis of the genes related to SCW formation in the first internodes.

To further validate the function of these DELLA-interacting NACs in cotton stem development, we employed the VIGS method to downregulate their expressions. The effects of *TRV:GhSND2s* treatment is shown in Figure 6. The expression levels of *GhSND2-1A, GhSND2-1D, GhSND2-2A, GhSND2-2D, GhSND2-3A*, and *GhSND2-3D* were all downregulated in the first internode of TRV:GhSND2s plants 15 DPI (Figure 6A). Meanwhile, lighter phloroglucinol staining intensity in the xylem area and phloem fibers was observed in the first internode of *ChSND2s*-silenced plants compared with TRV:00 plants (Figure 6B), indicating the knockdown of *GhSND2s* resulted in reduced lignification and SCW development in cotton stems. We further examined the expression levels of SCW-related genes and found that the knockdown of *GhSND2s* suppressed the expression of genes associated with cellulose (*CesA8*) and hemicellulose biosynthesis (*IRX14*), and lignin polymerization (*LAC4, PER4*, and *PER21*). Monolignol biosynthetic genes (such as *PAL, 4CL, CCoAOMT, COMT*, and *F5H*), however, had no differential expression levels between TRV:GhSND2s and TRV:00 cotton plants (Figure 6C), which was consistent with the report on *Arabidopsis* (Hussey et al., 2011). The VIGS of the other groups of DELLA-interacting NACs (GhFSNs and GhVNDs) also resulted in reduced SCW formation in cotton stems (Supplementary Figure 8). These results demonstrated that these DELLA-interacting NACs positively regulate the SCW formation in cotton stems.

## Discussion

SCW formation in stems is essential to plant growth and has a wealth of industrial applications (Kumar et al., 2016; Hofte and Voxeur, 2017; Zhong et al., 2019). This research represented an effort to study this important process with a rapid genetic method (VIGS) in a new system (the first internode of cotton). In this system, the effects of gene knockdown could be observed in a short period (<1 month), as exemplified in the silencing of DELLA and NAC genes. Cotton has a stem structure typical of woody plants. Cultivar cotton exhibits the characteristics of herbaceous plants, such as annual and shrub-like plants, because of artificial selection (Mcgarry et al., 2016). Considering their accessibility and readiness, the first internode of growing cotton may be an alternative model, other than *Arabidopsis* and poplar (Kumar et al., 2016; Zhong et al., 2019), for biological research on SCW (wood) formation.

Most dicots have DELLA proteins that belong to two clades (Van De Velde et al., 2017), such as cotton (Supplementary Figure 1). DELLAs in clade II were characterized by a conserved DGLLA motif instead of the DELLA motif in clade I. Rice DGLLA proteins SLRL1 and SLRL2 may suppress plant growth like DELLA protein (Itoh et al., 2005). In cotton stems, clade I DELLAs have dominant expression compared with clade II DELLAs (Supplementary Figure 2). Silencing of both clades of DELLA genes resulted in increased stem growth. The clade II DELLA genes showed lesser effects than those of clade I, and simultaneous silencing of these DELLA proteins had additive effects on promoting cotton growth (Figures 2C,D), indicating that the two clades of DELLA may have redundant functions in regulating the development of cotton stems. Although western blot only detected GhGAI1 degradation induced by GAs (Figure 1), we envisioned that both clade I and II DELLAs shared a similar mechanism to affect stem development in cotton.

DELLA proteins are the major repressor of GA signaling, and mediate GA response by interacting with a wealth of downstream regulators (Daviere and Achard, 2013). In SCW regulation, KNAT1 was reported to interact with DELLA protein and mediated GA promotion of SCW differentiation in *Arabidopsis*. DELLA proteins also repress the functions of NAC29/31 and MYB103L in SCW formation in rice. Here, we indicated that DELLA protein interacted with at least 10 NAC proteins, which were homologous to most switches that promote SCW formation. It can be predicted that there existed other SCW regulators that have not yet been identified, and DELLA proteins and their interactors constituted a transcriptional regulatory network mediating GA regulation of secondary cell walls in plants.

In addition to GAs, other phytohormones are involved in the formation of wood. Auxin plays a key role in regulating wood formation by affecting cambium activity and xylem development (Savidge, 1996; Uggla et al., 1996, 1998; Tuominen et al., 1997). Brassinosteroids are thought to be produced in procambial cells and trigger xylem precursor cells to induce xylem differentiation and regulate secondary cell wall biosynthesis (Milhinhos and Miguel, 2013; Yuan et al., 2019). Ethylene synthesis occurs in the apoplast of xylem elements; and ethylene participates, in a paracrine manner, in the control of the cambial stem cell pool size during secondary xylem formation (Pesquet and Tuominen, 2011). More and more studies have revealed a comprehensive view of DELLAs by identifying interactions with several types of TFs, which are considered to be central command systems that integrate various signals (Daviere and Achard, 2013). A previous study on *Arabidopsis* has revealed that DELLA interacts with ARFs, BZR1, and EIN3 (An et al., 2012; Bai et al., 2012; Oh et al., 2014) to mediate crosstalk between gibberellin and auxin, brassinosteroids, and ethylene, respectively. Therefore, DELLA may be a key component that integrates GA with other phytohormones in wood formation regulation.

## Data Availability Statement

The datasets presented in this study can be found in online repositories. The names of the repository/repositories and accession number(s) can be found below: https://www.ncbi.nlm.nih.gov/genbank/, PRJNA703439.

## Author Contributions

YWa and YX designed and wrote the manuscript. YWa, WY, LR, YDo, YQ, YDa, YWu, and XO performed experiments. YWa, WY, ZC, CW, YDa, YWu, and XO analyzed data. QS, JZ, YL, and AL discussed and corrected the manuscript. All authors contributed to the article and approved the submitted version.

## Conflict of Interest

The authors declare that the research was conducted in the absence of any commercial or financial relationships that could be construed as a potential conflict of interest.
