# Supplementary material for: DELLA-NAC Interactions Mediate GA Signaling to Promote Secondary Cell Wall Formation in Cotton Stem
Source: Front Plant Sci. 2021 Jul 9;12:655127. doi: 10.3389/fpls.2021.655127 (PMC8299300; doi:10.3389/fpls.2021.655127)
Supplement: Supplementary file 1 [file Data_Sheet_1.docx]

DELLA-NAC interactions mediate GA signaling to promote secondary cell wall formation in cotton stem

**Supplementary Table 1. Gene-specific primers used in this study**

| **Primer name** | **Primer sequence (5'-3')** |
| --- | --- |
| VIGS-GhGAI1A/D F | GACGAGTTATTAGCTGTTTTG |
| VIGS-GhGAI1A/D R | GTCGAACTCGATCAACAAAT |
| VIGS-GhGAI2A/D F | GATGAGCTTTTGGCGGTTTTG |
| VIGS-GhGAI2A/D R | GTTAAGTTCAGAGAGCATGCT |
| VIGS-GhGAI3A/D F | GATGGACTACTCGCCGGTGCT |
| VIGS-GhGAI3A/D R | CTCAGCGAACTCAGTTAGCAG |
| VIGS-GhGAI4A/D F | GATGGTTTTCTAGCCGGAGCT |
| VIGS-GhGAI4A/D R | GAGTAAACTTGGACAGCAGCG |
| VIGS-GhSND2-1A/D F | AACACAGCCTAGACAATGTG |
| VIGS-GhSND2-1A/D R | ACCCATCATGGACTGCAAAG |
| VIGS-GhSND2-2A/D F | AACTCAACCGAGACAATGCG |
| VIGS-GhSND2-2A/D R | TGAGCTGAGGTGGGCTTTCC |
| VIGS-GhSND2-3A/D F | CTCAACCTAGGCAATGCGGT |
| VIGS-GhSND2-3A/D R | CTTGTATCAGAGGCTAATCG |
| VIGS-GhVND4-1A/D F | GCAGCAACAACATACCATCA |
| VIGS-GhVND4-1A/D R | ATGCTTGGTGCGGTTGATTG |
| VIGS-GhVND4-2A/D F | CCTCATGACCCTACCTTTCA |
| VIGS-GhVND4-2A/D R | GTTGAGGACTGCAAAGTGCT |
| VIGS-GhVND4-3A/D F | ACCATTATCCCTGCAAGCAA |
| VIGS-GhVND4-3A/D R | TCCGTAAGGAACAACCGAGT |
| VIGS-GhVND4-4A/D F | TGCAAAATATGGCTTACCAC |
| VIGS-GhVND4-4A/D R | AAATGCTTCGACGGGTGGGT |
| VIGS-GhVND4-5A/D F | ACCAAACATCCCTATCTAAT |
| VIGS-GhVND4-5A/D R | AAATGCAGCCATTGGATTGC |
| VIGS-GhFSN1A/D F | TAACAACACGAGATTTGTCAT |
| VIGS-GhFSN1A/D R | GCCCTGAGTCATTGTTATAA |
| VIGS-GhFSN2A/D F | CAACAACGTTGATATCCCCAA |
| VIGS-GhFSN2A/D R | TGGACTCACAAACACCAGCAG |
| AD-GhGAI1A/D-GRAS F | catatgCTCGTTCATACATTAATGGCTTG |
| AD-GhGAI1A/D-GRAS R | atcgatTCAACTCAGCTCCTGAGTTAACTC |
| BD-GhVND4-3A F | cagaggaggacctgcatATGAATAACTTTACAAACGTTC |
| BD-GhVND4-3A R | cgctgcaggtcgacggatccTCACTTCCACAGGTCAATTTGA |
| BD-GhVND4-5D F | cagaggaggacctgcatATGAATGTACTTCCACAAGTTC |
| BD-GhVND4-5D R | cgctgcaggtcgacggatccTTACTTTGATTGGCGATATATC |
| BD-GhVND1-4D F | cagaggaggacctgcatATGGAATACTTATCAAAGCAAC |
| BD-GhVND1-4D R | cgctgcaggtcgacggatccTCATTGATCAAATATGCATATT |
| BD-GhFSN1A F | cagaggaggacctgcatATGCAAAGTTCATTTGGTATAT |
| BD-GhFSN1A R | cgctgcaggtcgacggatccTTATACACTAGCATTAACCACG |
| BD-GhFSN2D F | cagaggaggacctgcatATGTCAGAAGATATGAATCTAT |
| BD-GhFSN2D R | cgctgcaggtcgacggatccTTATACCGATAGATGGCATAAA |
| BD-GhSND2-1A/D F | cagaggaggacctgcatATGACTTGGTGCAATGACTGTA |
| BD-GhSND2-1A/D R | cgctgcaggtcgacggatccTCAAGGAATGAAAGACCCATCA |
| BD-GhSND2-2A/D F | cagaggaggacctgcatATGACATGGTGCAATAACCCAG |
| BD-GhSND2-2A/D R | cgctgcaggtcgacggatccCTATTTTCTTTCAACTCTCCCT |
| BD-GhSDN2-3A/D F | cagaggaggacctgcatATGACATGGTGCAATAACACG |
| BD-GhSDN2-3A/D R | cgctgcaggtcgacggatccTCAAAGTGATCTCCCTTTCCCT |
| BiFC-GhGAI1D F | ggggacaagtttgtacaaaaaagcaggcttcATGAAGAGAGATCATCAAGAAA |
| BiFC-GhGAI1D R | ggggaccactttgtacaagaaagctgggtcACTCAGCTCCTGAGTTAACTCA |
| BiFC-GhVND4-3A F | ggggacaagtttgtacaaaaaagcaggcttcATGAATAACTTTACAAACGTTC |
| BiFC-GhVND4-3A R | ggggaccactttgtacaagaaagctgggtcCTTCCACAGGTCAATTTGA |
| BiFC-GhVND4-5D F | ggggacaagtttgtacaaaaaagcaggcttcATGAATGTACTTCCACAAGTTC |
| BiFC-GhVND4-5D R | ggggaccactttgtacaagaaagctgggtcCTTTGATTGGCGATATATC |
| BiFC-GhVND1-4D F | ggggacaagtttgtacaaaaaagcaggcttcATGGAATACTTATCAAAGCAAC |
| BiFC-GhVND1-4D R | ggggaccactttgtacaagaaagctgggtcTTGATCAAATATGCATATT |
| BiFC-GhFSN1A F | ggggacaagtttgtacaaaaaagcaggcttcATGCAAAGTTCATTTGGTATAT |
| BiFC-GhFSN1A R | ggggaccactttgtacaagaaagctgggtcTACACTAGCATTAACCACG |
| BiFC-GhFSN2D F | ggggacaagtttgtacaaaaaagcaggcttcATGTCAGAAGATATGAATCTAT |
| BiFC-GhFSN2D R | ggggaccactttgtacaagaaagctgggtcTACCGATAGATGGCATAAA |
| BiFC-GhSND2-1A/D F | ggggacaagtttgtacaaaaaagcaggcttcATGACTTGGTGCAATGACTGTA |
| BiFC-GhSND2-1A/D R | ggggaccactttgtacaagaaagctgggtcAGGAATGAAAGACCCATCA |
| BiFC-GhSND2-2A/D F | ggggacaagtttgtacaaaaaagcaggcttcATGACATGGTGCAATAACCCAG |
| BiFC-GhSND2-2A/D R | ggggaccactttgtacaagaaagctgggtcTTTTCTTTCAACTCTCCCT |
| BiFC-GhSDN2-3A/D F | ggggacaagtttgtacaaaaaagcaggcttcATGACATGGTGCAATAACACG |
| BiFC-GhSDN2-3A/D R | ggggaccactttgtacaagaaagctgggtcAAGTGATCTCCCTTTCCCT |
| RT-GhGAI1A F | GGACCACCTCAACCCGATG |
| RT-GhGAI1A R | CGATGCGTTCGGCCAATTC |
| RT-GhGAI1D F | GGACCGCCTCAACCGGATA |
| RT-GhGAI1D R | CGATGCGTTCGGCCAATTG |
| RT-GhGAI2A F | CACCCACCAACGTTAAATCT |
| RT-GhGAI2A R | ACCACGGGACGAGTTGAAG |
| RT-GhGAI2D F | CACCCACCAACGTTAAATCC |
| RT-GhGAI2D R | ACCACGGGACGAGTTGAAT |
| RT-GhGAI3A F | TTCTTTAGAAGCTTGCAGGG |
| RT-GhGAI3A R | CCAATGGCTCGTGCCTTTCT |
| RT-GhGAI3D F | TTCTTTAGAAGCTTGCAGGA |
| RT-GhGAI3D R | CAATGGCTCGTGCCTTTCC |
| RT-GhGAI4A F | GTTCATCATGGATCCTGTAAG |
| RT-GhGAI4A R | GAATCTTCCTCCATTGCTGG |
| RT-GhGAI4D F | GTTCATCATGGATCCTGAAAC |
| RT-GhGAI4D R | GAATCTTCCTCCATTGCTGT |
| RT-GhSND2-1A F | GAAGAAAGGTTCACACCGACAC |
| RT-GhSND2-1A R | GCCAAGATGGTACTGGTGCATC |
| RT-GhSND2-1D F | GAGAGAAGCTCTCCTCCATCTTA |
| RT-GhSND2-1D R | CCATTCTCTCCTTCAATAGTAGGA |
| RT-GhSND2-2A F | ATCTGCGCAAGCTTCATCCGTT |
| RT-GhSND2-2A R | CCCGGCAACTTCTCTGGATGAG |
| RT-GhSND2-2D F | CATCCTGAGAAGTTGCCGGGAG |
| RT-GhSND2-2D R | TTCCTTGTCCCGGTTGTGTACG |
| RT-GhSND2-3A F | ACCGGTCATGAAGATGGAAGCG |
| RT-GhSND2-3A R | TACCGCATTGCCTAGGTTGAGC |
| RT-GhSND2-3D F | ACCGGTGATGAAGATGGAAGCG |
| RT-GhSND2-3D R | TACCGCATTGCCTAGGTTGAGT |
| RT-GhCesA4 F | CTCTCCTTGAGGTTCGAGCGTG |
| RT-GhCesA4 R | ACAACACACTTTCTCGACCGGG |
| RT-GhCesA7 F | GGTTGCTGCCCTTGTTTTGGAC |
| RT-GhCesA7 R | TCGCTTCCCCATTCGGTTTTGT |
| RT-GhCesA8 F | CATCCTTGCCTTGGACTACCCG |
| RT-GhCesA8 R | ATTCAAATGTCAGCATGGCCGC |
| RT-GhIRX9 F | GGACCTGTTTGCGATTCGTCAC |
| RT-GhIRX9 R | TTCTGTGAAGTGCCTTGGACCG |
| RT-GhIRX14 F | CACCAGAGCTACATCAGCCGTC |
| RT-GhIRX14 R | GGATCGGAACGGCGACGTATAG |
| RT-GhPAL F | GCTGTTGGGTCTGGGTTAGCTT |
| RT-GhPAL R | CTTGCTTTGGCTTCTGCAACGG |
| RT-Gh4CL F | CGACGTCGTAGCGCTTCCTTAT |
| RT-Gh4CL R | CTCGTGACCAAACCTTTGTGCG |
| RT-GhCCoAOMT F | AACACACTGTGGAATGGGTCGG |
| RT-GhCCoAOMT R | TCACCAACAGGGAGCATGCAAA |
| RT-GhCOMT F | GGCTGATGCAAAAGGCGATGTC |
| RT-GhCOMT R | AACATGTCACCCCCAACGTGAG |
| RT-GhF5H F | TCATGGATGTGATGTTCGGCGG |
| RT-GhF5H R | CGCCAACTCCTGCTGTACTCTC |
| RT-GhLAC4 F | ACTGCACCGTCCGACATTACAC |
| RT-GhLAC4 R | AGCCCAACCTGTTCTTAGCTGC |
| RT-GhPER4 F | CTTGCCCCGGTGTAGTCTCTTG |
| RT-GhPER4 R | CCACTATTCGCAGCAGCAAAGC |
| RT-GhPER21 F | CCACCATTGCTGCCTCAGCTTT |
| RT-GhPER21 R | CAGCATTGGGGCAACTTTGAGC |
| RT-GhACT F | TTGCAGACCGTATGAGCAAG |
| RT-GhACT R | ATCCTCCGATCCAGACACTG |

**Supplementary Table 2. GAI homologous genes identified in assembled cotton genomes**

| Homeologous group | G. raimondii (D5) | G. arboreum (A2) | G. hirsutum-Dt1 | G. hirsutum-At1 |
| --- | --- | --- | --- | --- |
| GoGAI1 | GrGAI1 | GaGAI1 | GhGAI1D | GhGAI1A |
|  | Gorai.001G089400 | Cotton_A_30811 | Gh_D07G0779 | Gh_A07G0717 |
| GoGAI2 | GrGAI2 | GaGAI2 | GhGAI2D | GhGAI2A |
|  | Gorai.002G177000 | Cotton_A_41295 | Gh_D01G1446 | Gh_A01G1242 |
| GoGAI3 | GrGAI3 | GaGAI3 | GhGAI3D | GhGAI3A |
|  | Gorai.010G067000 | Cotton_A_20431 | Gh_D06G0560 | Gh_A06G0504 |
| GoGAI4 | GrGAI4 | GaGAI4 | GhGAI4D | GhGAI4A |
|  | Gorai.009G021700 | Cotton_A_11125 | Gh_D05G0197 | Gh_A05G0135 |

Genes are named according to Hu’s report (Hu et al., 2011), and homeologs annotated in the genome sequencing projects for allotetraploid G. hirsutum (Dt1 and At1, https://phytozome.jgi.doe.gov/pz/portal.html#!info?alias=Org_Ghirsutum_er) and its extant diploid progenitors G. raimondii (D5) (Paterson et al., 2012) and G. arboreum (A2) (Li et al., 2014) are list in the same line.


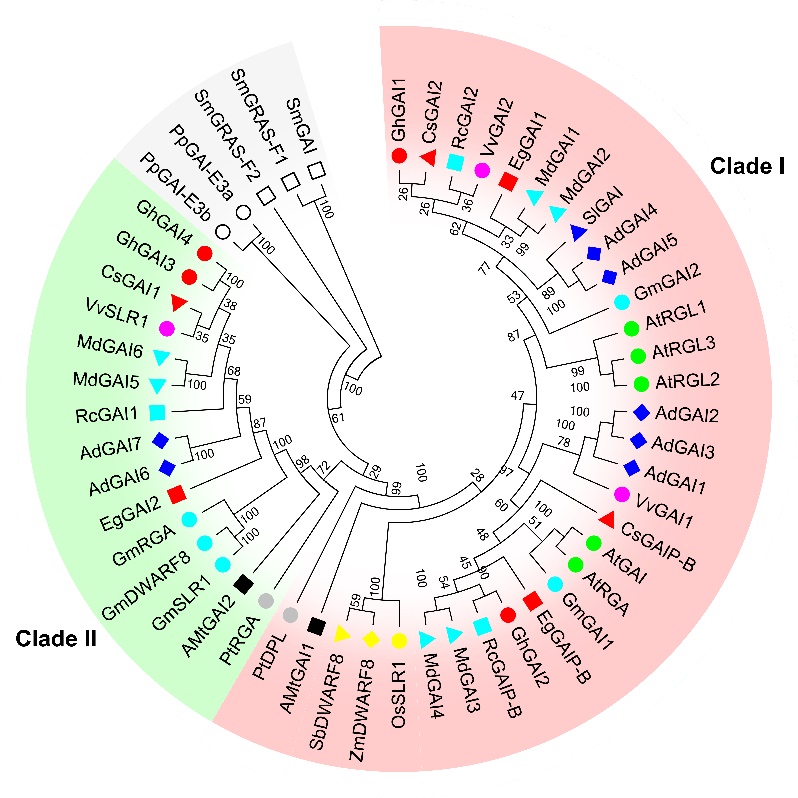


**Supplementary Figure 1. Phylogenetic analysis of DELLA proteins in seed plants.**

An unrooted phylogenetic tree of DELLA protein sequences from *Physcomitrella patens* (Pp), *Selaginella moellendorffii* (Sm), *Pinus tabuliformis* (Pt), *Amborella trichopoda* (Amt), *Oryza sativa* (Os), *Zea mays* (Zm), *Sorghum bicolor* (Sb), *Solanum lycopersicum* (Sl), *Actinidia deliciosa* (Ad), *Ricinus communis* (Rc), *Glycine max* (Gm), *Malus domestica* ( Md), *Arabidopsis thaliana* (At), *Gossypium hirsutum* (Gh), *Citrus sinensis* (Cs), *Eucalyptus grandis* (Eg), *Vitis vinifera* (Vv). The phylogenetic tree was constructed using DELLA protein sequences and the neighbor-joining (NJ) method in MEGA 5.0 software, and the bootstrap test was performed with 1,000 iterations. Clade I is marked red, clade II is marked green, the rest is marked gray.


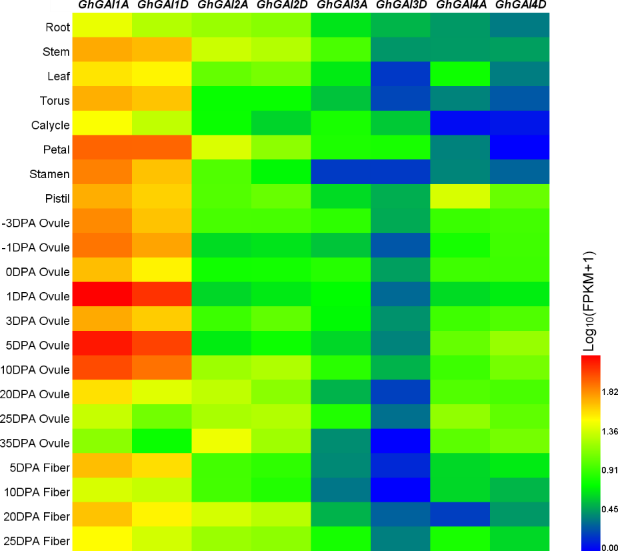


**Supplementary Figure 2. Transcript levels of *DELLA* genes in various upland cotton tissues.**

The gene expression levels (FPKM) in root, stem, leaf, torus, calycle, petal,stamen,pistil, ovules, and fibers of various days post anthesis (DPA) are converted to Log10(FPKM + 1) and illustrated in the heat map. Transcript levels are inferred from the transcriptomic data the CottonFGD collections (<https://cottonfgd.org/>).


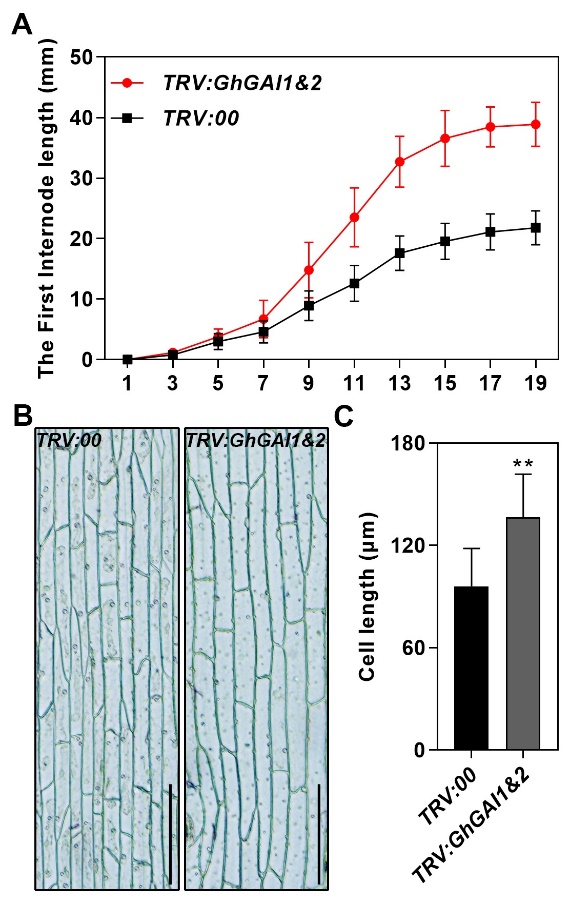


**Supplementary Figure 3.** **Silencing of *GhGAI1A/D* and *GhGAI2A/D* promote cell elongation in the first internode of cotton plants.**

(A) Recording the elongate process of the first internode of control and *GhGAI1&2*- silenced cotton plants. n≥10. (B) A comparative anatomical evaluation of the lengthways-sections of the first internode in middle area from control and *GhGAI1&2*- silenced cotton plants at 15 days post-infiltration. Bar=100 μm. (C) Measurement and statistical analysis of cell length of the first internode in control and *GhGAI1&2*- silenced cotton plants at 15 days post infiltration. n≥100. * and ** indicate a significant difference compared to control by student’s t test with P-values of 0.05 and 0.01, respectively. Error bars are SD.


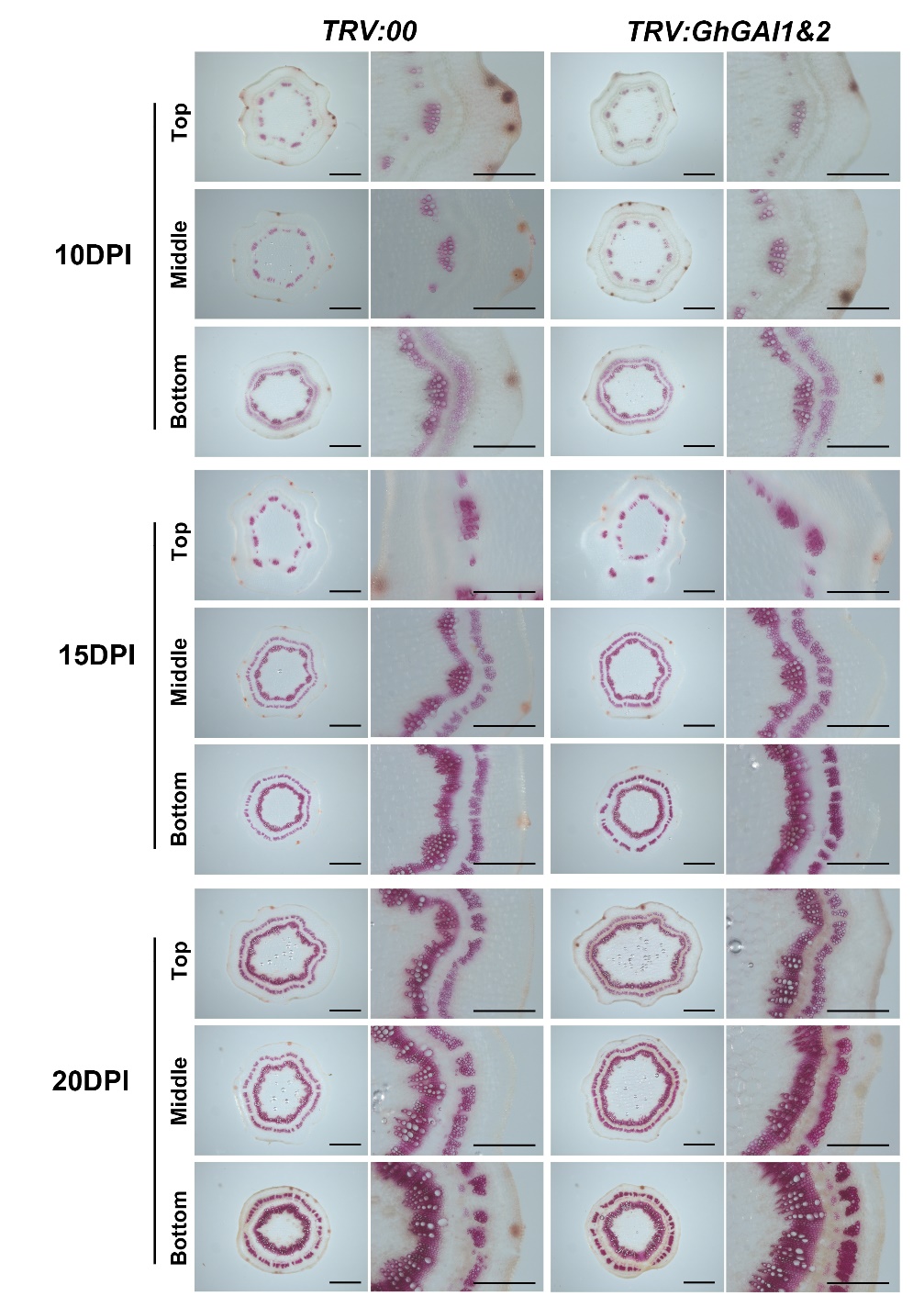


**Supplementary Figure 4.** Cross-sections of the first internode from control and *GhGAI1&2*- silenced cotton plants at 10,15 and 20 days post infiltration were stained with phloroglucinol. Bar=1mm or 500μm (amplification).


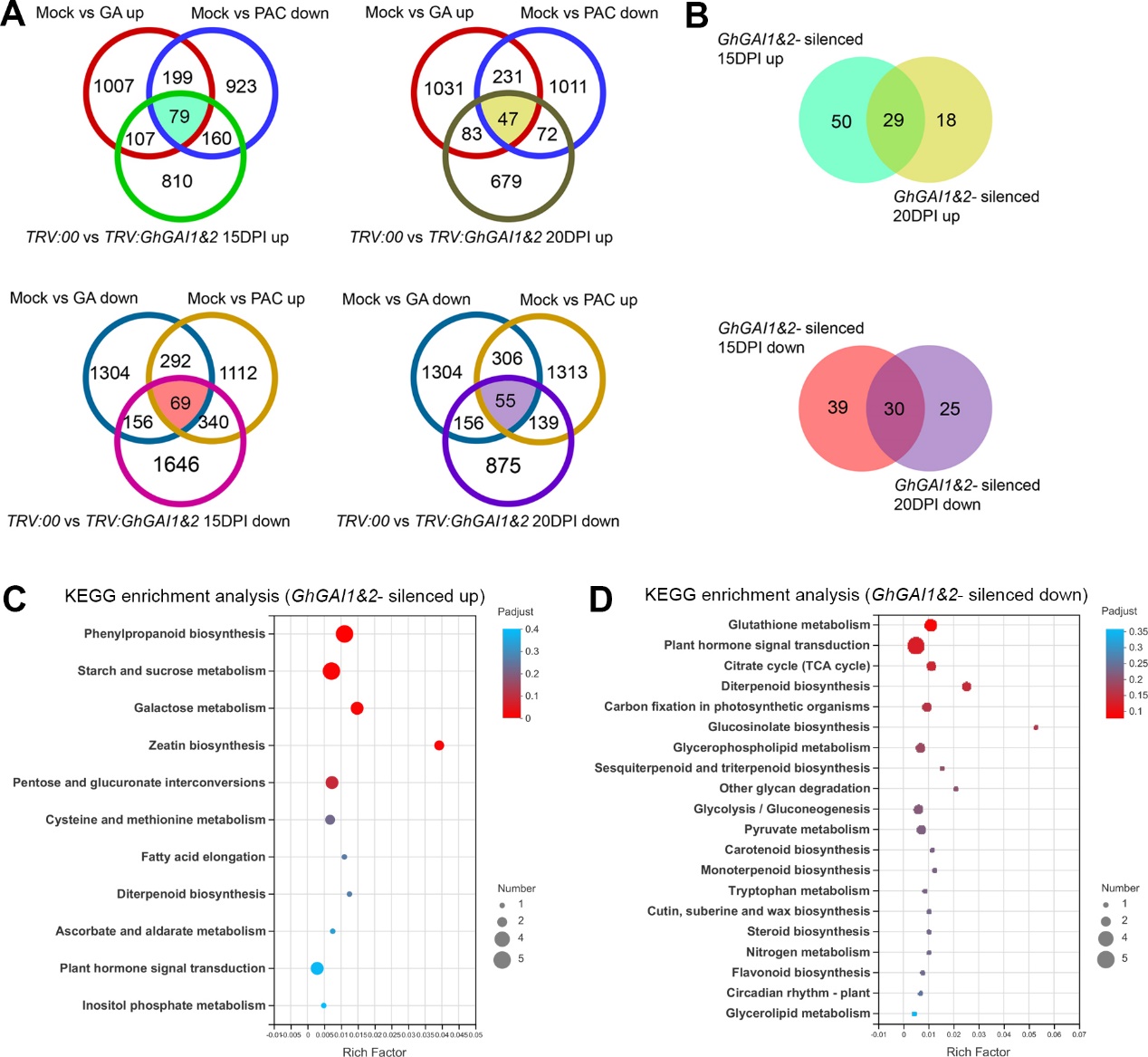


**Supplementary Figure 5. RNA-Seq analysis of the differentially expressed in *GhGAI1&2*-** **silenced plants**

Total RNAs were isolated from the first internode of 10-d-old wild type treated with mock, 100 μM GA, 20 μM PAC for 8 day, as well as TRV:00 and GhGAI1&2- silenced plants at 15 & 20 DPI. (A) Venn diagrams show significant overlap among differentially expressed gene sets of GA-treated, PAC-treated, and *GhGAI1&2-* silenced at 15 DPI or 20 DPI. (B) Venn diagrams show that genes regulated by GhGAI1&2. DEG sets with same fill color in (A). (C) Up-regulated DEG under *GhGAI1&2-* silenced enriched KEGG pathway scatterplot. (D) Down-regulated DEG under *GhGAI1&2-* silenced enriched KEGG pathway scatterplot.


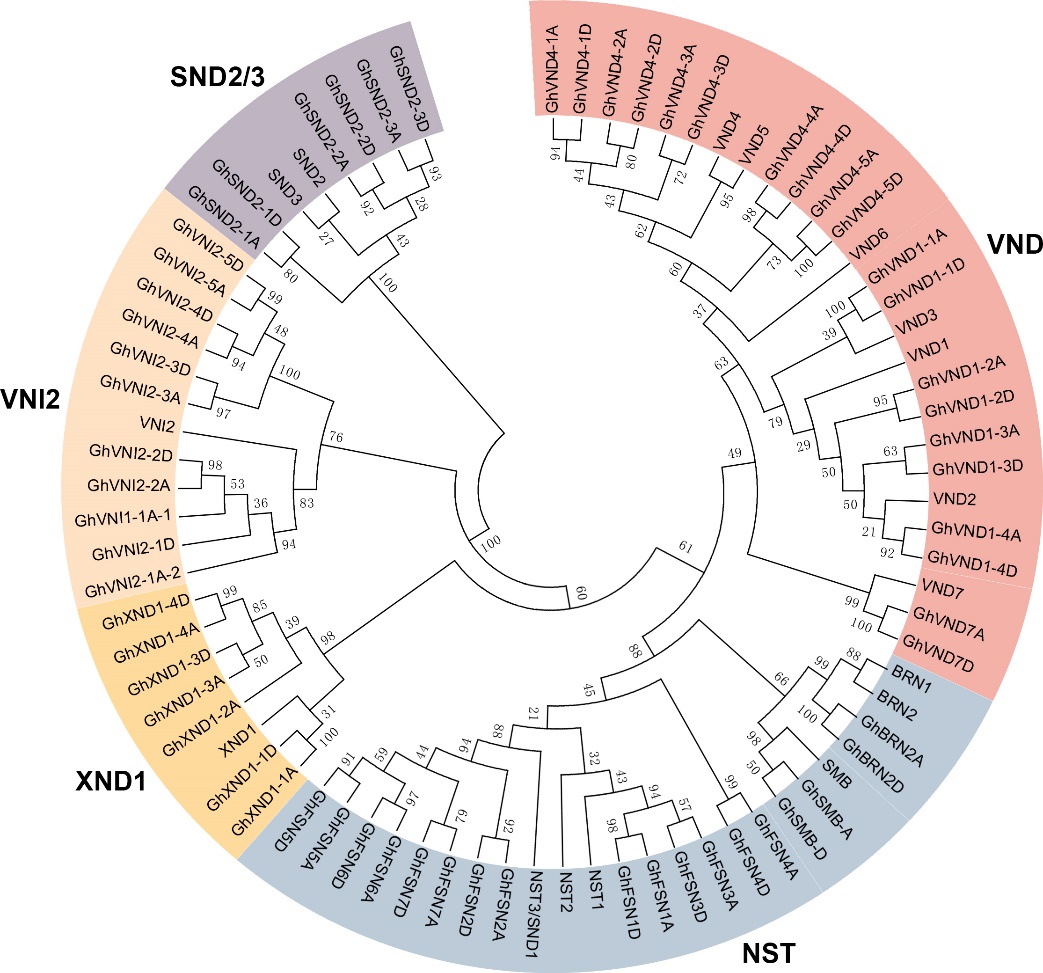


**Supplementary Figure 6. Phylogenetic analysis of SCW-related NAC proteins in upland cotton and** **Arabidopsis.**

The phylogenetic tree was constructed using SCW-related NAC protein sequences and the neighbor-joining (NJ) method in MEGA 5.0 software, and the bootstrap test was performed with 1,000 iterations.

**Supplementary Table 3. SCW-related NAC homologous genes in cotton**

| **Arabidopsis homologs** | **Gene Name** | **Gene ID** | **Gene_description** | **FPKM value** | | | | | | |
| --- | --- | --- | --- | --- | --- | --- | --- | --- | --- | --- |
|  |  |  |  | **Mock** | **GA** | **PAC** | **TRV:00 15DPI** | **TRV:GhGAI1&2 15DPI** | **TRV:00 20DPI** | **TRV:GhGAI1&2 20DPI** |
| VND4,5,6 | GhVND4-1A | Gh_A03G1986 | NAC 007 | 2.26 | 2.43 | 1.93 | 1.95 | 1.65 | 1.21 | 1.52 |
|  | GhVND4-1D | Gh_D03G1542 | NAC 007 | 3.31 | 1.75 | 3 | 2.3 | 1.58 | 2.03 | 1.5 |
|  | GhVND4-2A | Gh_A08G1552 | NAC 007 | 1.59 | 0.8 | 1.76 | 0.88 | 1.01 | 0.77 | 1 |
|  | GhVND4-2D | Gh_D08G1858 | NAC 007 | 0.37 | 0.38 | 0.87 | 0.58 | 0.31 | 0.79 | 0.51 |
|  | GhVND4-3A | Gh_A12G1990 | NAC 007 | 1.64 | 1.41 | 2.45 | 2.03 | 0.6 | 1.76 | 1.73 |
|  | GhVND4-3D | Gh_D12G2169 | NAC 007 | 1.46 | 1.07 | 1.84 | 1.83 | 1.44 | 1.27 | 1.76 |
|  | GhVND4-4A | Gh_A07G0349 | NAC 007 | 0.74 | 0.39 | 0.61 | 0.54 | 0.96 | 0.45 | 0.4 |
|  | GhVND4-4D | Gh_D07G0411 | NAC 007 | 0.4 | 0.23 | 0.41 | 1.02 | 0.22 | 0.58 | 0.81 |
|  | GhVND4-5A | Gh_A03G0506 | NAC 007 | 4.5 | 3.02 | 3.58 | 4.69 | 5.72 | 4.93 | 2.96 |
|  | GhVND4-5D | Gh_D03G1021 | NAC 007 | 2.35 | 2.49 | 2.78 | 2.12 | 3.08 | 2.15 | 2.49 |
| VND1,2,3 | GhVND1-1A | Gh_A02G1625 | vascular related NAC-domain protein 1 | 0.09 | 0.21 | 0.78 | 0.19 | 0.11 | 0.17 | 0.32 |
|  | GhVND1-1D | Gh_D03G0096 | vascular related NAC-domain protein 1 | 0.78 | 0.66 | 1 | 0.23 | 0.1 | 0.42 | 0.29 |
|  | GhVND1-2A | Gh_A11G0386 | vascular related NAC-domain protein 1 | 1.41 | 0.55 | 0 | 0.36 | 1.97 | 2.15 | 1.41 |
|  | GhVND1-2D | Gh_D11G0448 | vascular related NAC-domain protein 1 | 1.68 | 2.99 | 0.53 | 0.2 | 1.91 | 0.78 | 2.94 |
|  | GhVND1-3A | Gh_A11G1635 | vascular related NAC-domain protein 1 | 1.34 | 0.93 | 1.97 | 1.15 | 0.74 | 0.55 | 0.95 |
|  | GhVND1-3D | Gh_D11G1793 | vascular related NAC-domain protein 1 | 0 | 0 | 0 | 0 | 0.1 | 0.33 | 0 |
|  | GhVND1-4A | Gh_A12G0557 | vascular related NAC-domain protein 1 | 2.05 | 3 | 1.71 | 1.69 | 1.86 | 2.8 | 5.11 |
|  | GhVND1-4D | Gh_D12G0573 | vascular related NAC-domain protein 1 | 0.98 | 2.43 | 1.89 | 1.69 | 1.54 | 1.93 | 3.56 |
| VND7 | GhVND7A | Gh_A13G1020 | vascular related NAC-domain protein 7 | 0.4 | 2.27 | 0.51 | 0.6 | 0.33 | 0.44 | 0.12 |
|  | GhVND7D | Gh_D13G1271 | vascular related NAC-domain protein 7 | 0.18 | 0.51 | 0.21 | 0.09 | 0.15 | 0.04 | 0 |
| BRN2 | GhBRN2A | Gh_A08G2042 | NAC domain containing protein 70 | 0 | 0 | 0 | 0 | 0.09 | 0 | 0.04 |
|  | GhBRN2D | Gh_D08G2434 | NAC domain containing protein 70 | 0 | 0 | 0 | 0 | 0.05 | 0 | 0.04 |
| SMB | GhSMB-A | Gh_A08G2289 | NAC (No Apical Meristem) domain transcriptional regulator superfamily protein | 0 | 0 | 0 | 0 | 0 | 0 | 0 |
|  | GhSMB-D | Gh_D08G2447 | NAC (No Apical Meristem) domain transcriptional regulator superfamily protein | 0 | 0 | 0 | 0 | 0 | 0 | 0 |
| NST1~3 | GhFSN1A | Gh_A12G1049 | NAC (No Apical Meristem) domain transcriptional regulator superfamily protein | 10.15 | 9.35 | 3.2 | 8.26 | 7.56 | 7.03 | 10.18 |
|  | GhFSN1D | Gh_D12G1169 | NAC (No Apical Meristem) domain transcriptional regulator superfamily protein | 13.97 | 16.47 | 5.85 | 9.48 | 8.23 | 12.33 | 13.02 |
|  | GhFSN2A | Gh_A12G2179 | NAC (No Apical Meristem) domain transcriptional regulator superfamily protein | 3 | 2.21 | 1.46 | 1.02 | 2.9 | 1.98 | 4.46 |
|  | GhFSN2D | Gh_D12G2359 | NAC (No Apical Meristem) domain transcriptional regulator superfamily protein | 2.01 | 1.95 | 0.91 | 0.51 | 1.34 | 0.8 | 2 |
|  | GhFSN3A | Gh_A11G0915 | NAC (No Apical Meristem) domain transcriptional regulator superfamily protein | 8.45 | 13.87 | 4.95 | 4.62 | 6.95 | 12.62 | 13.26 |
|  | GhFSN3D | Gh_D11G1062 | NAC (No Apical Meristem) domain transcriptional regulator superfamily protein | 15.87 | 27.36 | 10.13 | 9.69 | 15.74 | 20.88 | 23.84 |
|  | GhFSN4A | Gh_A07G1226 | NAC (No Apical Meristem) domain transcriptional regulator superfamily protein | 2.3 | 4.54 | 0.5 | 2.25 | 3.87 | 3.06 | 3.23 |
|  | GhFSN4D | Gh_D07G1330 | NAC (No Apical Meristem) domain transcriptional regulator superfamily protein | 2.21 | 4.94 | 0.51 | 1.63 | 2.4 | 2.56 | 3.45 |
|  | GhFSN5A | Gh_A08G0961 | NAC (No Apical Meristem) domain transcriptional regulator superfamily protein | 2.16 | 2.54 | 1.65 | 2.98 | 3.24 | 2.61 | 2.91 |
|  | GhFSN5D | Gh_D08G1172 | NAC (No Apical Meristem) domain transcriptional regulator superfamily protein | 1.47 | 1.56 | 1.36 | 1.72 | 1.97 | 1.34 | 1.74 |
|  | GhFSN6A | Gh_A02G0977 | NAC (No Apical Meristem) domain transcriptional regulator superfamily protein | 7.31 | 5.69 | 8.27 | 7.96 | 7.38 | 7.12 | 8.8 |
|  | GhFSN6D | Gh_D03G0775 | NAC (No Apical Meristem) domain transcriptional regulator superfamily protein | 4.93 | 3.41 | 4.87 | 7.5 | 6.98 | 7.21 | 6.53 |
|  | GhFSN7A | Gh_A09G2371 | NAC (No Apical Meristem) domain transcriptional regulator superfamily protein | 6.53 | 8.38 | 4.96 | 6.33 | 7.05 | 6.11 | 7.29 |
|  | GhFSN7D | Gh_D09G1785 | NAC (No Apical Meristem) domain transcriptional regulator superfamily protein | 2.06 | 7.26 | 2.65 | 3.12 | 3.04 | 2.56 | 2.77 |
| XND1 | GhXND1-1A | Gh_A05G3322 | xylem NAC domain 1 | 0 | 0 | 0 | 0 | 0 | 0 | 0 |
|  | GhXND1-1D | Gh_D04G0278 | xylem NAC domain 1 | 3.3 | 0.97 | 3.23 | 2.09 | 1.82 | 3.11 | 0.39 |
|  | GhXND1-2A | Gh_A13G0575 | xylem NAC domain 1 | 0.37 | 0.45 | 0.36 | 0.56 | 0.09 | 0.62 | 0.83 |
|  | GhXND1-3A | Gh_A03G1512 | xylem NAC domain 1 | 7.97 | 7.67 | 4.09 | 5.14 | 6.59 | 5.88 | 10.02 |
|  | GhXND1-3D | Gh_D02G1981 | xylem NAC domain 1 | 3.24 | 5.45 | 1.71 | 4.14 | 4.87 | 4.92 | 7.49 |
|  | GhXND1-4A | Gh_A12G0118 | xylem NAC domain 1 | 1.92 | 2.55 | 3.04 | 6.41 | 4.54 | 1.88 | 2.68 |
|  | GhXND1-4D | Gh_D12G0132 | xylem NAC domain 1 | 4.62 | 1.37 | 5.64 | 4.81 | 5.59 | 1.6 | 3.16 |
| VNI2 | GhVNI2-1A-1 | Gh_A03G1333 | NAC domain containing protein 83 | 1.98 | 1.93 | 1.8 | 1.56 | 1.28 | 0.58 | 0.93 |
|  | GhVNI2-1A-2 | Gh_A03G1332 | NAC domain containing protein 83 | 23.99 | 21.97 | 24.54 | 18.91 | 28.81 | 26.3 | 30.65 |
|  | GhVNI2-1D | Gh_D02G1769 | NAC domain containing protein 83 | 31.51 | 28.67 | 33.62 | 23.39 | 26.5 | 25.44 | 25.85 |
|  | GhVNI2-2A | Gh_A04G0868 | NAC domain containing protein 83 | 55.48 | 50.15 | 43.14 | 48.25 | 67.22 | 54.95 | 55.8 |
|  | GhVNI2-2D | Gh_D04G1368 | NAC domain containing protein 83 | 71.28 | 70.67 | 49.88 | 32.21 | 39.67 | 56.84 | 48.25 |
|  | GhVNI2-3A | Gh_A05G2928 | NAC domain containing protein 83 | 2.62 | 1.81 | 2.83 | 0.73 | 1.64 | 0.25 | 0.28 |
|  | GhVNI2-3D | Gh_D04G0712 | NAC domain containing protein 83 | 6.25 | 9.53 | 9.75 | 1.17 | 1.15 | 1.44 | 1.52 |
|  | GhVNI2-4A | Gh_A10G0627 | NAC domain containing protein 83 | 1.53 | 0.69 | 2 | 0.46 | 0.77 | 2.13 | 1.42 |
|  | GhVNI2-4D | Gh_D10G0772 | NAC domain containing protein 83 | 3.8 | 3.31 | 4.12 | 2.32 | 3.36 | 3.92 | 3.01 |
|  | GhVNI2-5A | Gh_A07G2132 | NAC domain containing protein 83 | 6.19 | 5.03 | 5.38 | 4.89 | 5.3 | 4.19 | 3.09 |
|  | GhVNI2-5D | Gh_D07G2341 | NAC domain containing protein 83 | 6.09 | 5.72 | 7.04 | 3.05 | 3.48 | 4.49 | 3.8 |
| SND2/3 | GhSND2-1A | Gh_A12G1285 | NAC domain containing protein 73 | 19.66 | 19.39 | 11.27 | 10.17 | 12.93 | 21.67 | 23.95 |
|  | GhSND2-1D | Gh_D12G1407 | NAC domain containing protein 73 | 15.87 | 24 | 10.03 | 17.11 | 6.53 | 19.73 | 33.27 |
|  | GhSND2-2A | Gh_A05G1339 | NAC domain containing protein 10 | 6.65 | 6.78 | 3.42 | 6.68 | 5.1 | 8.23 | 8.23 |
|  | GhSND2-2D | Gh_D05G1508 | NAC domain containing protein 73 | 5.67 | 4.38 | 3.77 | 5.1 | 4.46 | 6.07 | 6.53 |
|  | GhSDN2-3A | Gh_A06G2028 | NAC domain containing protein 73 | 6.31 | 8.81 | 4.75 | 4.59 | 3.9 | 6.83 | 7.96 |
|  | GhSND2-3D | Gh_D06G1417 | NAC domain containing protein 73 | 0 | 0 | 0 | 0 | 0 | 0 | 0 |


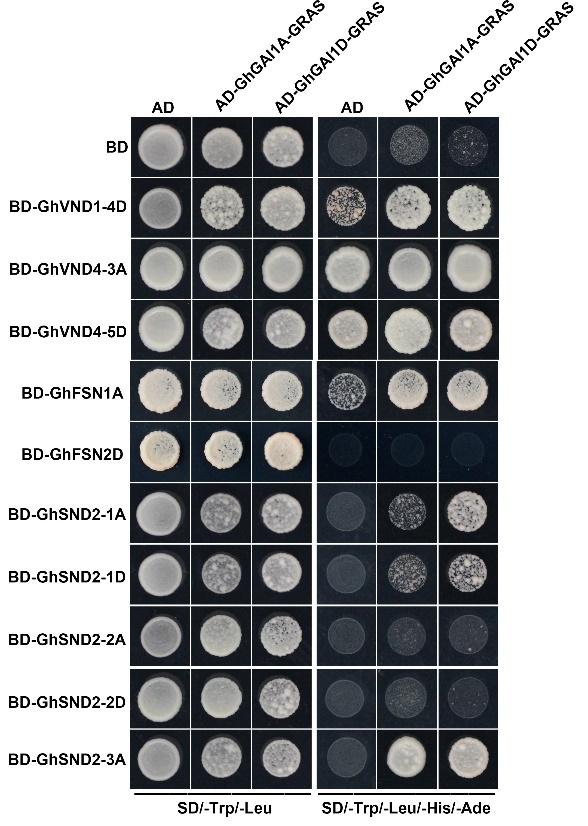


**Supplementary Figure 7. Yeast two-hybrid assays show the interactions between GhGAI1A/D and****SCW-related NAC proteins.**


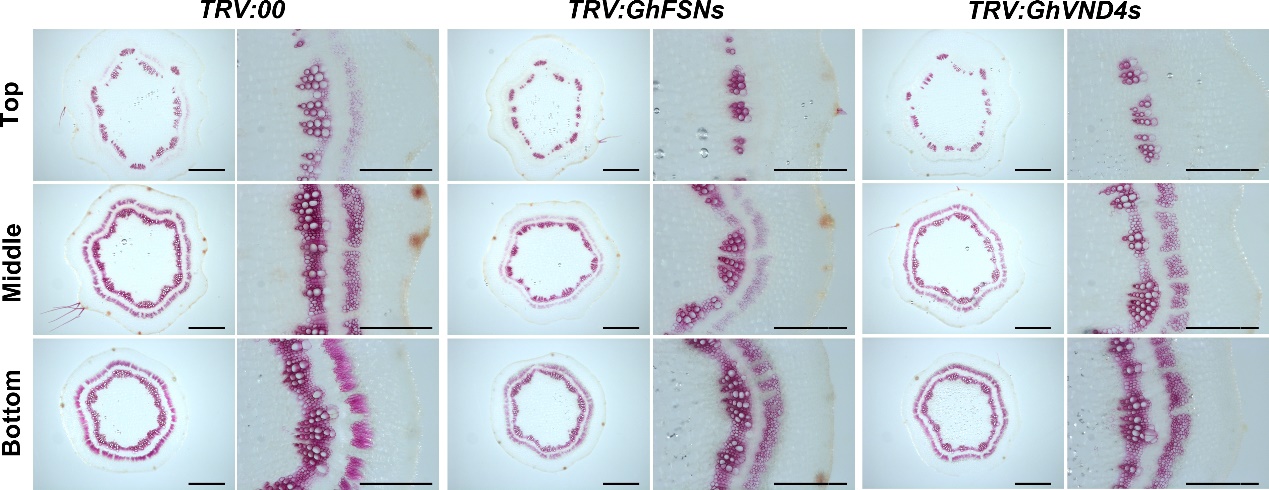


**Supplementary Figure 8. Functional characterization of GhFSNs and GhVND4s by VIGS analysis.**

Cross-sections of the first internode from control and *GhFSNs*- silenced and *GhVND4s*- silenced cotton plants at 15 days post infiltration were stained with phloroglucinol. Bar=1mm or 500μm (amplification).

Hu, M.-y., Luo, M., Xiao, Y.-h., Li, X.-b., Tan, K.-l., Hou, L., . . . Pei, Y. (2011). Brassinosteroids and Auxin Down-Regulate DELLA Genes in Fiber Initiation and Elongation of Cotton. *Agricultural Sciences in China, 10*(8), 1168-1176. doi:10.1016/s1671-2927(11)60107-7

Li, F., Fan, G., Wang, K., Sun, F., Yuan, Y., Song, G., . . . Yu, S. (2014). Genome sequence of the cultivated cotton Gossypium arboreum. *Nat Genet, 46*(6), 567-572. doi:10.1038/ng.2987

Paterson, A. H., Wendel, J. F., Gundlach, H., Guo, H., Jenkins, J., Jin, D., . . . Schmutz, J. (2012). Repeated polyploidization of Gossypium genomes and the evolution of spinnable cotton fibres. *Nature, 492*(7429), 423-427. doi:10.1038/nature11798
